# A positron emission tomography study of nigro-striatal dopaminergic mechanisms underlying attention: implications for ADHD and its treatment

**DOI:** 10.1093/brain/awt263

**Published:** 2013-10-25

**Authors:** Natalia del Campo, Tim D. Fryer, Young T. Hong, Rob Smith, Laurent Brichard, Julio Acosta-Cabronero, Samuel R. Chamberlain, Roger Tait, David Izquierdo, Ralf Regenthal, Jonathan Dowson, John Suckling, Jean-Claude Baron, Franklin I. Aigbirhio, Trevor W. Robbins, Barbara J. Sahakian, Ulrich Müller

**Affiliations:** 1 Department of Psychiatry, University of Cambridge, Addenbrooke’s Hospital, Cambridge CB2 0QQ, UK; 2 Behavioural and Clinical Neuroscience Institute, Department of Psychology, University of Cambridge, Downing Site, Cambridge CB2 3EB, UK; 3 Wolfson Brain Imaging Centre, Addenbrooke’s Hospital, Cambridge CB2 0QQ, UK; 4 Department of Clinical Neurosciences, University of Cambridge, Addenbrooke’s Hospital, Cambridge CB2 0QQ, UK; 5 German Centre for Neurodegenerative Diseases, 39120 Magdeburg, Germany; 6 Adult ADHD Service, Cambridgeshire and Peterborough NHS Foundation Trust, Addenbrooke’s Hospital, Cambridge CB2 0QQ, UK; 7 Department of Clinical Pharmacology, University of Leipzig, 04107 Leipzig, Germany; 8 Inserm U894, Centre Hospitalier Sainte-Anne, Sorbonne Paris Cité, Paris 75014, France

**Keywords:** attention deficit/hyperactivity disorder, ^18^F-fallypride PET, dopamine, methylphenidate, sustained attention

## Abstract

Through the combined use of ^18^F-fallypride positron emission tomography and magnetic resonance imaging this study examined the neural mechanisms underlying the attentional deficits associated with attention deficit/hyperactivity disorder and their potential reversal with a single therapeutic dose of methylphenidate. Sixteen adult patients with attention deficit/hyperactivity disorder and 16 matched healthy control subjects were positron emission tomography and magnetic resonance imaging scanned and tested on a computerized sustained attention task after oral methylphenidate (0.5 mg/kg) and placebo administration in a within-subject, double-blind, cross-over design. Although patients with attention deficit/hyperactivity disorder as a group showed significant attentional deficits and reduced grey matter volume in fronto-striato-cerebellar and limbic networks, they had equivalent D_2_/D_3_ receptor availability and equivalent increases in endogenous dopamine after methylphenidate treatment to that observed in healthy control subjects. However, poor attentional performers drawn from both the attention deficit/hyperactivity disorder and the control groups had significantly reduced left caudate dopamine activity. Methylphenidate significantly increased dopamine levels in all nigro-striatal regions, thereby normalizing dopamine levels in the left caudate in low performers. Behaviourally, methylphenidate improved sustained attention in a baseline performance-dependent manner, irrespective of diagnosis. This finding was accompanied by an equally performance-dependent effect of the drug on dopamine release in the midbrain, whereby low performers showed reduced dopamine release in this region. Collectively, these findings support a dimensional model of attentional deficits and underlying nigro-striatal dopaminergic mechanisms of attention deficit/hyperactivity disorder that extends into the healthy population. Moreover, they confer midbrain dopamine autoreceptors a hitherto neglected role in the therapeutic effects of oral methylphenidate in attention deficit/hyperactivity disorder. The absence of significant case–control differences in D_2_/D_3_ receptor availability (despite the observed relationships between dopamine activity and attention) suggests that dopamine dysregulation *per se* is unlikely to be the primary cause underlying attention deficit/hyperactivity disorder pathology in adults. This conclusion is reinforced by evidence of neuroanatomical changes in the same set of patients with attention deficit/hyperactivity disorder.

## Introduction

Attention deficit/hyperactivity disorder (ADHD) is a neurodevelopmental disorder characterized by symptoms of inattention and/or hyperactivity/impulsivity. Despite its declining prevalence with age, ADHD symptoms in some affected patients persist into adulthood, affecting ∼2.5% of the adult population (Simon *et al.*, 2009). The clinical effectiveness of stimulants such as methylphenidate in the treatment of ADHD suggests a putative role for both dopamine and noradrenaline in the manifestation of the disorder (Castells *et al.*, 2011). However, despite decades of genetic, clinical and neuroimaging research, the precise neurobiological mechanisms underlying the disorder and its treatment remain poorly understood.

Long-standing cognitive theories of ADHD postulate a deficiency in top–down cognitive control processes underpinning attention and executive function deficits (Barkley, 1997; Nigg, 2000; Bush *et al.*, 2005; Kuntsi *et al.*, 2006; Paloyelis *et al.*, 2007 for review see Del Campo *et al.*, 2012). Providing support to these theories, one of the best replicated findings in ADHD is that of reduced grey matter volume in the catecholamine-rich fronto-striato-cerebellar circuits known to subserve these cognitive functions (Seidman *et al.*, 2005; Nakao *et al.*, 2011). Notwithstanding the growing evidence on fronto-striatal dysfunction and the efficacy of stimulant drugs in the clinical management of patients with ADHD, the precise role of dopamine in the pathophysiology of the disorder is still unclear. Indeed, *in vivo* nuclear imaging techniques such as PET and single photon emission computed tomography (SPECT) have often yielded inconsistent findings regarding the state of the dopamine system in this patient population (Del Campo *et al.*, 2011*a*). A recent set of case–control PET studies in adult medication-naïve patients found that ADHD is associated with reduced dopamine transporter and dopamine D_2_/D_3_ receptor availability in selected brain regions of the left hemisphere, including the nucleus accumbens, caudate and midbrain (Volkow *et al.*, 2007*a*, *b*, 2009). Although these impressive studies had substantial numbers of patients, there was considerable overlap with the control population in terms of both dopamine receptor and transporter availabilities, implying that the diagnostic utility of these markers is limited.

The binding competition between D_2_/D_3_ receptor radioligands and endogenous dopamine allows measuring changes in dopamine levels following a pharmacological challenge, for example a psychostimulant. Acute administration of methylphenidate would be expected to increase endogenous dopamine, leading to a reduction of D_2_/D_3_ receptor radioligand binding through competitive displacement. To date there are two studies that have used this imaging paradigm to examine the endogenous tone of the dopamine system in patients with ADHD. One study in adolescent patients with ADHD found that oral methylphenidate-induced increases in dopamine concentrations in the right striatum were greater in patients showing poor performance on a computerized continuous performance test relative to high performing patients (Rosa-Neto *et al.*, 2005). A subsequent study of intravenous methylphenidate effects in adult patients with ADHD showed blunted dopamine responses to methylphenidate in the caudate in patients compared with control subjects, although there was no concurrent objective measure of cognitive performance (Volkow *et al.*, 2007*b*).

Although it is possible that the state of the striatal dopamine system changes with age in ADHD, in this dopamine receptor PET imaging study we sought to resolve the above discrepancy by investigating the effects of oral methylphenidate on both endogenous dopamine levels and sustained attention in adult patients with ADHD compared with healthy matched control subjects using a within-subject, counter-balanced placebo-controlled design. High resolution MRI scans were also acquired and analysed for case–control differences in grey matter volume to allow, for the first time in the ADHD literature, the interpretation of the PET results in the light of potential brain tissue abnormalities. The full set of MRI data, including also diffusion tensor imaging data, will be reported in more detail elsewhere.

As a methodological innovation, which further distinguishes the current study from previous ones, we used the co-registered high resolution magnetic resonance images to accurately localize PET signals within the striatum and the midbrain. We employed a model of functional rather than purely anatomical subdivisions of the striatum (Haber *et al.*, 2000) that has been increasingly used to optimize the analysis of PET studies (Kegeles *et al.*, 2010). This model is based on evidence that cortico-striatal networks follow a ventro-medial to dorsolateral gradient, with activity along this gradient modulating limbic, cognitive and motor processing (Martinez *et al.*, 2003). Through its connections with the striatum, the midbrain operates as an interface for the dynamic processing of striatal function. According to preclinical evidence, stimulant-induced increases in endogenous dopamine levels trigger negative feedback mechanisms that inhibit dopamine neuron firing (Bunney and Achajanian, 1976). These negative feedback mechanisms are mediated by somato-dendritic D_2_/D_3_ autoreceptors located on midbrain dopamine neurons, which play a key role in regulating dopamine synthesis and release by acting as potent inhibitors in the presence of high dopamine concentrations (Mercuri *et al.*, 1992, 1997; Sesack *et al.*, 1994; Khan *et al.*, 1998). How the therapeutic effects of methylphenidate might depend on the afferent control of midbrain dopamine neurons has been unexplored to date. This question is particularly relevant because findings for both juvenile and adult ADHD have implicated functional and structural midbrain abnormalities in the pathophysiology of the disorder (Ernst *et al.*, 1999; Jucaite *et al.*, 2005; Volkow *et al.*, 2009; Romanos *et al.*, 2010), also consistent with experimental animal models of ADHD (Leo *et al.*, 2003).

To investigate nigro-striatal mechanisms underlying ADHD and its treatment with methylphenidate, we used PET imaging of ^18^F-fallypride, which has a higher affinity than ^11^C-raclopride (the radioligand of choice in all previous dopamine receptor imaging studies in ADHD) and is thus better suited for simultaneous measurement of D_2_/D_3_ receptor availability in striatal regions and the midbrain (Riccardi *et al.*, 2006*a*, *b*; Cropley *et al.*, 2008; Slifstein *et al.*, 2010). Through estimation of non-displaceable ^18^F-fallypride binding potential (BP_ND_), the following questions regarding the neurochemical underpinnings of ADHD were addressed.

First, do patients with ADHD have reduced D_2_/D_3_ receptor availability in the striatum and the midbrain compared with healthy volunteers? Second, how is D_2_/D_3_ receptor availability in these regions associated with inattention, a core feature of the disorder (Biederman *et al.*, 2000; Nigg, 2005)? Third, how does a therapeutic oral dose of methylphenidate affect D_2_/D_3_ receptor availability in the striatum and the midbrain?, and fourth, how are the effects on receptor availability related to drug-induced changes on performance on a task of sustained attention previously reported to be sensitive to single oral doses of this drug (Turner *et al.*, 2005)? Finally, are the observed drug effects on receptor availability quantitatively different in patients with ADHD and healthy volunteers?

The latter question is especially topical (Sahakian and Morein-Zamir, 2007) because methylphenidate has been reported to augment several aspects of cognition in healthy subjects (Rapoport *et al.*, 1978, 1980; Strauss *et al.*, 1984; Koelega, 1993; Elliott *et al.*, 1997; Mehta *et al.*, 2000) similar to those improved in ADHD (Mehta *et al.*, 2004; Turner *et al.*, 2005; Clark *et al.*, 2007; Spencer *et al.*, 2007). The effects of methylphenidate and related stimulant drugs in healthy individuals have been shown to be baseline-dependent; i.e. those subjects with relatively low performance levels exhibit greater benefit after the drug (Naylor *et al.*, 1985; Rogers *et al.*, 1999; Mehta *et al.*, 2000; Turner *et al.*, 2003; Clatworthy *et al.*, 2009). These findings can be interpreted in the light of a hypothesized inverted U-shaped function relating dopamine and noradrenaline activity to performance (Robbins and Sahakian, 1979). Of course, it is also possible that the cognitive enhancing effects in patients may similarly be dependent on baseline-dependent considerations.

According to previous reports of altered brain tissue volume and dysregulated dopamine neurotransmission in ADHD, we hypothesized that patients with ADHD would show reduced grey matter volume and D_2_/D_3_ receptor availability in selected brain regions involved in dopamine-modulated circuits (Volkow *et al.*, 2007*b*, 2009). We also hypothesized that methylphenidate would increase endogenous dopamine levels in both ADHD and healthy control subjects, improving attention in patients with ADHD and possibly also in some healthy volunteers as a function of baseline performance. In accordance with previous evidence linking abnormalities in the left caudate with attentional deficits, we expected dopamine dysregulation in this region to be specifically associated with impaired sustained attention on the computerized cognitive test.

## Materials and methods

### Subjects

Sixteen male adult patients with ADHD and 16 control subjects matched for gender, age and IQ were enrolled in the study after meeting inclusion criteria and providing written informed consent. The study was approved by the Cambridge Research Ethics Committee, The UK Administration of Radioactive Substances Advisory Committee and was formally exempted from clinical trial status by the UK Medicines and Healthcare Regulatory Agency.

Patients were recruited from the Adult ADHD Research Clinic Cambridge and through the Attention Deficit Disorder Information and Support Service (ADDISS), UK. Diagnosis was contingent upon six of nine DSM-IV (Diagnostic and Statistical Manual of Mental Disorders, fourth edition) inattention and/or hyperactivity/impulsivity criteria being met during childhood and the previous 6 months (1994). Clinical assessments were performed by two psychiatrists specialized in adult ADHD (J.D., U.M.) as described previously (Chamberlain *et al.*, 2007). Of the 16 patients, 15 met criteria for ‘combined type’ and one for ‘inattentive type’. Seven patients had a history of prescribed medication with methylphenidate, and one had previously been exposed to atomoxetine through participation in a past research study. The remaining patients (*n = *8) were ADHD medication-naïve. Patients on methylphenidate treatment at the time of scanning were asked to discontinue their medication at least 3 days before each PET session.

All participants underwent an extended clinical interview using the Mini International Neuropsychiatric Inventory (MINI) (Sheehan *et al.*, 2010). Exclusion criteria were the presence of axis-I disorders or any major neurological or internal disease, left handedness, verbal IQ <90 [National Adult Reading Test (NART)] (Nelson, 1982) and current cigarette smoking and/or drug abuse, as controlled by urine screening (Euromed, Drug Screen Card DOA-154-731, 5072KAB). Participants were asked to abstain from drinking alcoholic or caffeine-containing drinks for 12 h before each testing session.

### Experimental design and cognitive assessment

**Figure 1:**
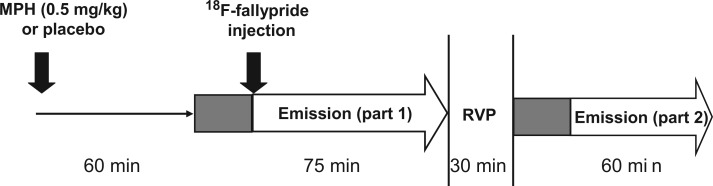
Time line for PET and RVP data acquisition after capsule administration. Grey boxes indicate 15 min transmission scans.

Participants were enrolled into a randomized, double-blind placebo-controlled cross-over design, consisting of two ^18^F-fallypride PET scans at least a week apart and one MRI scan. On each PET session, subjects were administered a capsule containing either 0.5 mg/kg of methylphenidate or placebo 75 min before the ^18^F-fallypride injection (Fig. 1). The dose of 0.5 mg/kg was chosen to be within the therapeutic range used in the clinic: according to the NICE 2008 guidelines regarding the diagnosis and treatment of patients with ADHD, starting methylphenidate doses of 3 × 5 mg or 3 × 10 mg and a dose limit of 100 mg per day are recommended. A dose of 0.5 mg/kg equals 40 mg for an 80 kg patient, representing thus a medium dose for a single dose study. At 0, 2 and 4 h after capsule administration, blood samples were collected for determination of methylphenidate plasma concentrations (Supplementary material) to assess compliance with drug discontinuation in medicated patients and account for between-subject differences in methylphenidate plasma levels. Cardiovascular measures were regularly monitored throughout the study.

On both PET sessions, participants performed the Rapid Visual Information Processing (RVP) task from CANTAB (Cambridge Automated Neuropsychological Testing Battery, www.camcog.com). RVP primarily measures sustained attention, defined as the ability to maintain attention on a series of numerical stimuli over a period of time, to detect infrequent targets (Coull *et al.*, 1996). The task was administered during a scanning break 2.5 h after capsule intake (Fig. 1), coinciding with the estimated peak effectiveness of methylphenidate (Challman and Lipsky, 2000). Performance on the RVP task was measured by the non-parametric signal detection parameter A’ (discriminative sensitivity) (McNicol, 1972). The selection of RVP A’ to measure attentional performance in this study is supported by evidence that this parameter (i) is a suitable ADHD endophenotype (Gau and Huang, 2013); (ii) is associated with activity in neural networks including frontal and striatal regions (Lawrence *et al.*, 2003); and (iii) is sensitive to the enhancing effects of drugs known to be effective in ADHD, previously reported in both patients with ADHD (Turner *et al.*, 2005) and healthy control subjects (Crockett *et al.*, 2010; Supplementary material). Response latencies were also measured. To minimize practice effects, all subjects performed the task once before their first study session.

All participants completed the adult ADHD self-rating scale (v1.1), a short self-rating test addressing the severity of ADHD symptoms in adults consistent with DSM-IV criteria (Kessler *et al.*, 2005).

### Imaging

#### Magnetic resonance acquisition

Magnetic resonance images were acquired for all subjects on a Siemens Trio 3 T system (Siemens Medical Systems) with gradient coils capable of 45 mT/m and 200 T/m/s slew rate. A 12-channel total-imaging matrix head-coil was used to transmit/receive radio-frequency magnetic resonance signals. T_1_-weighted anatomical scans were acquired using a 3D MP-RAGE pulse sequence with the following parameters: repetition time/echo time/inversion time* = *2300 ms/2.98 ms/900 ms, flip angle 9°, 176 slices, 256 × 256 matrix size, 1 × 1 × 1 mm^3^ voxel size, echo spacing* = *7.1 ms, bandwidth* = *240 Hz per pixel. Images were manually aligned to the anterior commissure (AC) – posterior commissure (PC) line. A T_2_-weighted image was also acquired for each subject (echo time* = *104 ms, 27 slices, 0.7 × 0.7 × 4 mm^3^ voxel size).

#### Positron emission tomography acquisition

^18^F-fallypride PET data were acquired in 3D mode on a GE Advance scanner (General Electric Medical Systems). Before ^18^F-fallypride injection, a 15 min transmission scan was acquired. ^18^F-fallypride was injected intravenously over 30 s, with a mean activity of 103.5 MBq (range: 52.2–119.8 MBq), and a fallypride mass of 1.3 ± 0.7 nmols.

From the start of the ^18^F-fallypride injection PET emission data were continuously acquired for 75 min in 55 frames (Fig. 1). After a 30 min break, subjects were repositioned in the scanner and after another 15-min transmission scan, a second set of ^18^F-fallypride data were acquired over the next hour. Images were reconstructed using the PROMIS 3D filtered back-projection algorithm (Kinahan and Rogers, 1989) into 2.34 × 2.34 × 4.25 mm voxels.

#### Magnetic resonance imaging preprocessing and grey matter volume analysis

All MPRAGE scans were manually aligned to the AC–PC line (MR_AC-PC_). Before analysis, volumes were preprocessed, and then spatially normalized and segmented into grey and white matter tissue using the unified segmentation model in SPM5 (Ashburner and Friston, 2005). Statistical case–control differences in tissue volume were assessed through a non-parametric, permutation inference method using Cambridge Brain Analysis v1.3.2 (CAMBA; http://www-bmu.psychiatry.cam.ac.uk/software/) (Bullmore *et al.*, 1999). This methodology confers a number of advantages, notably the enhancement of sensitivity and the control for type I errors. For each between-group comparison, the statistical threshold of *β < *0.05 was applied. Results were corrected for multiple comparisons by controlling the family-wise error rate and setting the number of false positive clusters expected under the null hypothesis to *<*1. Clusters showing significant between-group differences were described in terms of the Automated Anatomical Labelling (AAL) template image (where voxels/region* > *20) (Tzourio-Mazoyer *et al.*, 2002).

#### Positron emission tomography realignment and co-registration to magnetic resonance imaging

Images from each PET scan were realigned and co-registered to the MR_AC-PC_ using SPM2 (www.fil.ion.ucl.ac.uk/spm) as previously described (Del Campo *et al.*, 2011*b*).

#### Region of interest delineation

**Figure 2:**
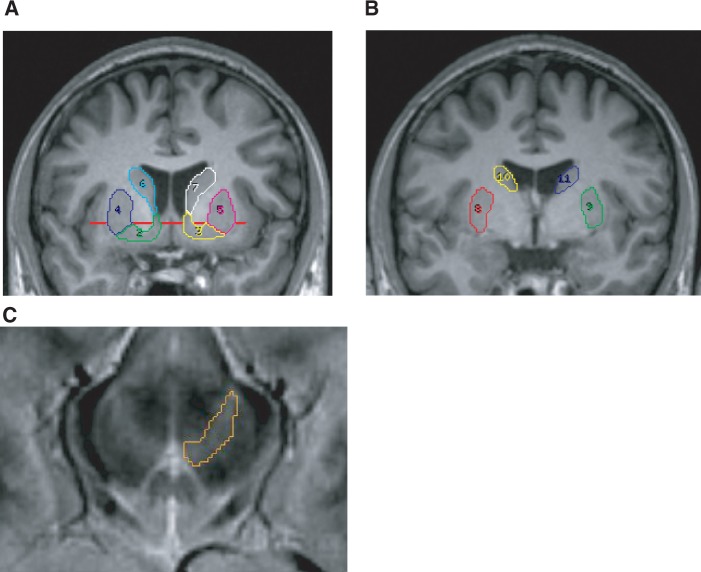
(**A** and **B**) Coronal planes through an AC–PC aligned MPRAGE image showing striatal regions of interest. (**A**) Ventral striatum (2, 3), pre-commissural dorsal putamen (4, 5), pre-commissural dorsal caudate (6, 7); (**B**) post-commissural putamen (8, 9) and post-commissural caudate (10, 11). The red line helped to divide the pre-commissural caudate and putamen into ventral and dorsal. (**C**) Midbrain region of interest defined on a T_2_ scan co-registered to the MR_AC-PC_.

For each subject, regions of interest were manually drawn on coronal planes of the MR_AC-PC_ with Analyze 7.0 (AnalyzeDirect) as previously described (Mawlawi *et al.*, 2001; Martinez *et al.*, 2003; Del Campo *et al.*, 2011*b*). The following striatal subregions were defined bilaterally: ventral striatum, pre-commissural dorsal putamen, pre-commissural dorsal caudate, post-commissural putamen and post-commissural caudate (Fig. 2).

These subregions allowed classification of the striatum into the following functional modules: ventral striatum; associative striatum, comprising pre-commissural dorsal caudate, post-commissural caudate and pre-commissural dorsal putamen and sensorimotor striatum, consisting of the post-commissural putamen (Haber *et al.*, 2000; Joel and Weiner, 2000; Haber, 2003). Evidence suggests that through their involvement in different cortico-striatal networks, these modules are functionally distinct, with the ventral striatum being implicated in emotion, motivation and reward-guided behaviours, the associative striatum in cognition, and the sensorimotor striatum in motor function (Martinez *et al.*, 2003). The aggregate of bilateral ventral striatum, associative striatum and sensorimotor striatum is henceforth referred to as total striatum.

Bilateral midbrain regions of interest including the substantia nigra and the ventral tegmental area were delineated on transverse planes of the MR_AC-PC_-coregistered T_2_-weighted image as described elsewhere (Foster *et al.*, 2008). To allow BP_ND_ values to be calculated using reference tissue modelling, a bilateral cerebellar region was drawn on the MR_AC-PC_.

#### Regional quantification of binding potential

Regional BP_ND_ was estimated by fitting the simplified reference tissue model (Gunn *et al.*, 1997) to region of interest time activity curves. The cerebellar time activity curve between 75–120 min post-injection was estimated by fitting an exponential function from 50 min onwards. To calculate mean BP_ND_ for associative striatum, bilateral regions and total striatum, BP_ND_ values for the constituent regions of interest were weighted in proportion to region of interest volume. Changes in receptor availability following methylphenidate relative to placebo were estimated by BP_ND_ % change:
(1)
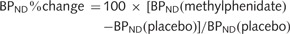



### Statistical analysis

Group effects on ADHD self-rating scale scores and both group and methylphenidate effects on task performance and BP_ND_ were tested using univariate and repeated measures analysis of variance (ANOVAs) with group and medication status as between-subject factors. Where assumptions of sphericity were violated, degrees of freedom were corrected by applying Greenhouse-Geisser corrections. Significant differences were further examined using *post hoc t*-tests or, where appropriate, the equivalent versions for non-parametric data (Wilcoxon signed-rank test and Mann-Whitney U test).

Pearson correlations and where appropriate Spearman’s rank correlations were used to describe relationships between non-imaging measures and both BP_ND_ and BP_ND_ % change. Where relevant, methylphenidate plasma concentrations were partialled out. Relationships were investigated for left and right hemispheres separately, in-line with previous observations of lateralization differences in dopaminergic parameters in the healthy population (Larisch *et al.*, 1998; Vernaleken *et al.*, 2007) as well as in patients with ADHD (Volkow *et al.*, 2007*a*, *b*, 2009). To allow assessment of group differences, correlation coefficients were transformed into z-scores. To control for the overall type 1 error rate, stepwise linear regression models were also used to establish the relative contributions of ^18^F-fallypride BP_ND_ to attentional performance variability, both at baseline and following methylphenidate. BP_ND_ measures of all regions of interest were entered into the models. An alpha level of 0.05 was chosen as the statistical threshold.

## Results

### Sample characteristics and cognitive performance

Demographic characteristics of the sample and mean scores on psychological measures are shown in Table 1. Both groups were matched for age and IQ. Patients with ADHD had significantly higher ADHD self-rating scale scores (*P* ≤ 0.01) and performed significantly worse on the RVP A’ signal detection parameter [main effect of group: *F*(1,29)* = *8.31, *P = *0.007; placebo data only: *F*(1,29)* = *9.89, *P = *0.004]. There were no between session practice effects on task performance (*P > *0.1).

**Table 1:** Subject characteristics

	ADHD patients (*n* = 16)	Controls (*n* = 16)	*F*-value	*P*-value
Age, years	30.3 (7.4)	28.9 (6.0)	0.3	0.57
NART	112.0 (9.2)	115.7 (4.4)	2.1	0.16
Adult Self Report Scale				
Total	54.1 (7.9)	25.9 (6.7)	68.4	**<0.001**
Inattention	28.1 (4.8)	14.5 (3.6)	48.3	**<0.001**
Hyperactivity	26.1 (4.8)	11.4 (4.8)	43.1	**<0.001**
RVP performance on placebo				
A’	0.92 (0.05)	0.97 (0.03)	9.9	**0.004**
Mean latency, ms	424 (59)	373 (40)	3.0	0.09

Values are mean (SD).

NART = National Adult Reading Test.

Group comparisons were carried out using ANOVAs, significant *P-*values (<0.05) are in bold.

### Grey matter volume

**Figure 3:**
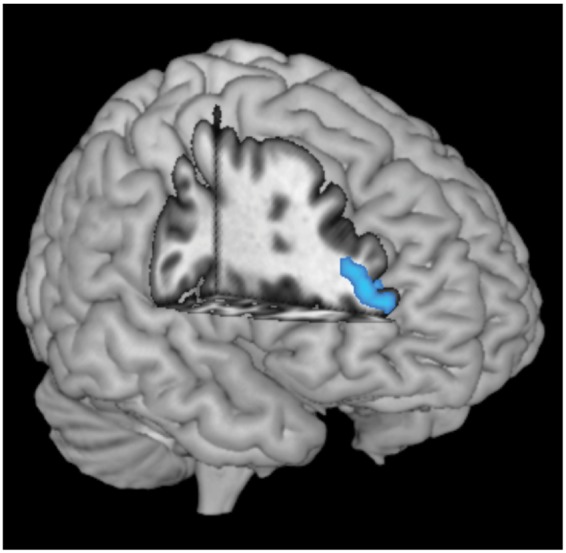
Cluster of greatest grey matter volume reduction in patients with ADHD compared with control subjects located in the left middle frontal gyrus, overlaid on a rendered standardized brain template.

No group differences were found in total intracranial volume and total grey matter volume (*P > *0.1). However, patients with ADHD showed significantly reduced grey matter volume in left prefrontal cortical areas (middle frontal cortex, inferior portions of the orbitofrontal cortex and gyrus rectus), and in bilateral putamen, amygdala, hippocampus, fusiform, insula and cerebellum (Table 2 and Fig. 3).

**Table 2:** Regions of decreased grey matter density in ADHD patients compared with controls

Cluster	Region name (AAL)	Location of peak (*x*, *y*, *z*) [mm]			Voxels	*F*
**GM 1** 503 vox	**Cerebellum crus II R**	**40**	**−66**	**−48**	**35**	**3.86**
	Cerebellum VIIB R	38	−66	−48	70	3.79
	Cerebellum VIII R	22	−74	−48	392	3.66
**GM 2** 2089 vox	Superior frontal gyrus (orbital portion) L	−16	16	−20	55	3.29
	Inferior frontal gyrus (pars triangularis) L	−40	26	2	71	4.11
	Inferior frontal orbital gyrus L	−26	32	−12	64	3.51
	Olfactory cortex L	−12	8	−18	49	3.76
	Gyrus rectus L	−6	38	−20	65	4.16
	Insula L	−38	0	−8	127	3.80
	**Hippocampus L**	**−22**	**−12**	**−12**	**297**	**4.59**
	Parahippocampal gyrus L	−28	−24	−24	133	3.74
	Amygdala L	−26	−4	−14	81	3.78
	Fusiform gyrus L	−28	−30	−20	173	3.86
	Putamen L	−28	2	−6	92	3.51
	Middle temporal gyrus L	−46	−2	−16	57	4.39
	Inferior temporal gyrus L	−38	−20	−28	23	2.54
	Cerebellum IV, V L	−14	−54	−24	184	3.11
	Cerebellum VI L	−14	−58	−24	213	3.52
	Vermis IV, V	−2	−60	−14	23	2.55
**GM 3** 692 vox	Hippocampus R	30	−30	−10	107	3.36
	Parahippocampal gyrus R	26	−34	−12	106	3.31
	Lingual gyrus R	28	−48	−6	28	3.42
	Fusiform gyrus R	38	−22	−26	106	4.05
	**Cerebellum IV, V R**	**26**	**−38**	**−24**	**166**	**4.25**
	Cerebellum VI R	36	−46	−32	144	3.57
**GM 4** 749 vox	Insula R	40	−8	2	114	3.43
	**Hippocampus R**	**34**	**−6**	**−18**	**56**	**5.51**
	Amygdala R	30	−4	−18	79	4.04
	Putamen R	34	−2	−6	202	3.89
**GM 5** 197 vox	**Middle frontal gyrus**	**−28**	**40**	**20**	**231**	**6.53**

Regional labels are clusters of the Automated Anatomical Labelling (AAL). Locations are in the stereotactic space of the MNI. Cluster peaks are highlighted in bold.

GM = grey matter.

For clarity, regional PET and behavioural results (and the relationship between both) are subsequently presented split by experimental session (placebo then methylphenidate). Supporting whole-brain voxel-wise PET results are presented and discussed in the Supplementary material.

### Placebo results

#### Regional analysis of BP_ND_ on placebo

**Figure 4:**
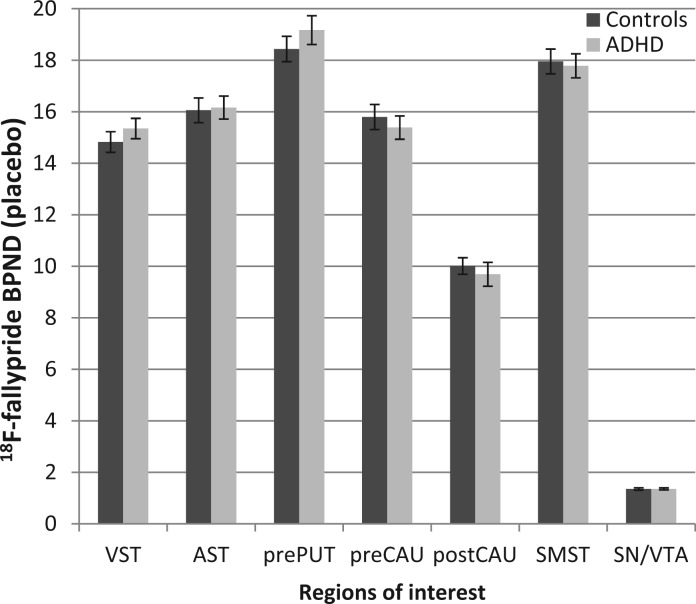
Regional variation in ^18^F-fallypride binding potential (BP_ND_) on placebo in healthy control subjects and ADHD patients. Error bars denote standard error of the mean. VST = ventral striatum; AST = associative striatum; prePUT = pre-commissural putamen; preCAU = pre-commissural caudate; postCAU = post-commissural caudate; SMST = sensorimotor striatum; SN/VTA = substantia nigra/ventral tegmental area.

The mean BP_ND_ values were not significantly different between patients with ADHD and control subjects across all regions (either unilaterally or bilaterally), with bilateral values for the latter shown in Fig. 4. Regional BP_ND_ values pooled across all subjects are shown in Table 3. BP_ND_ values on placebo were in the same range as those previously reported in a study examining test–retest variability of baseline ^18^F-fallypride BP_ND_ across the same brain regions (Cropley *et al.*, 2008) (Supplementary material).

**Table 3:** ^18^F-fallypride BP_ND_ at baseline and after methylphenidate, pooled across all subjects

		BP_ND_ (Placebo)			BP_ND_ (MPH)			BP_ND_ % change (MPH relative to placebo)		
Region		Left	Right	Bilateral	Left	Right	Bilateral	Left	Right	Bilateral
Striatum		16.3 (1.6)	16.4 (1.6)	16.4 (1.6)	15.3 (1.4)	15.4 (1.5)	15.4 (1.5)	**−6.2 (5.8)**	**−5.1 (5.9)**	**−5.9 (5.5)**
VST		15.3 (1.5)	14.9 (1.7)	15.1 (1.5)	14.4 (1.6)	14.5 (1.8)	14.5 (1.6)	**−5.6 (7.8)**	**−2.2 (10.1)**	**−4.0 (7.4)**
AST		16.1 (1.8)	16.2 (1.8)	16.1 (1.8)	15.1 (1.6)	15.2 (1.6)	15.2 (1.6)	**−5.4 (7)**	**−5.5 (5.5)**	**−5.4 (5.9)**
	Pre-PUT	18.6 (2.1)	18.9 (2.1)	18.8 (2.0)	17.5 (1.8)	17.7 (1.9)	17.6 (1.8)	**−5.8 (7.5)**	**−6.5 (6.1)**	**−6.2 (5.9)**
	Pre-CAU	15.7 (2.0)	15.5 (1.8)	15.6 (1.8)	14.8 (1.7)	14.8 (1.7)	14.8 (1.7)	**−4.8 (7.5)**	**−4.6 (6.3)**	**−4.8 (6.2)**
	Post-CAU	9.9 (1.6)	9.9 (1.7)	9.9 (1.5)	9.2 (1.5)	9.3 (1.6)	9.2 (1.5)	**−6.4 (10)**	−5.6 (10.3)	**−6.1 (9.1)**
SMST	Post-PUT	17.8 (2.0)	17.9 (1.8)	17.9 (1.8)	16.4 (1.7)	16.5 (1.7)	16.5 (1.7)	**−7.5 (7.2)**	**−7.7 (6.4)**	**−7.6 (6.1)**
SN/VTA		1.4 (0.2)	1.3 (0.2)	1.4 (0.2)	1.3 (0.2)	1.2 (0.2)	1.2 (0.2)	**−7.2 (10.5)**	**−8.0 (11.0)^a^**	**−7.8 (8.5)**

Data are mean (SD).

AST = associative striatum; CAU = caudate; post = post-commissural; pre = pre-commissural; PUT = putamen; SMST = sensorimotor striatum; SN/VTA = substantia nigra/ventral tegmental area; VST = ventral striatum.

Levels of significance of BP_ND_ % change were assessed with paired-samples *t*-tests and, where appropriate, with ^a^ Wilcoxon Signed ranks test. BP_ND_ changes significant below Bonferroni corrected level of *P* < 0.003 for left and right regions and *P* < 0.005 for bilateral regions are in bold.

#### Relationship between ADHD self-rating scale and regional BP_ND_ on placebo

**Figure 5:**
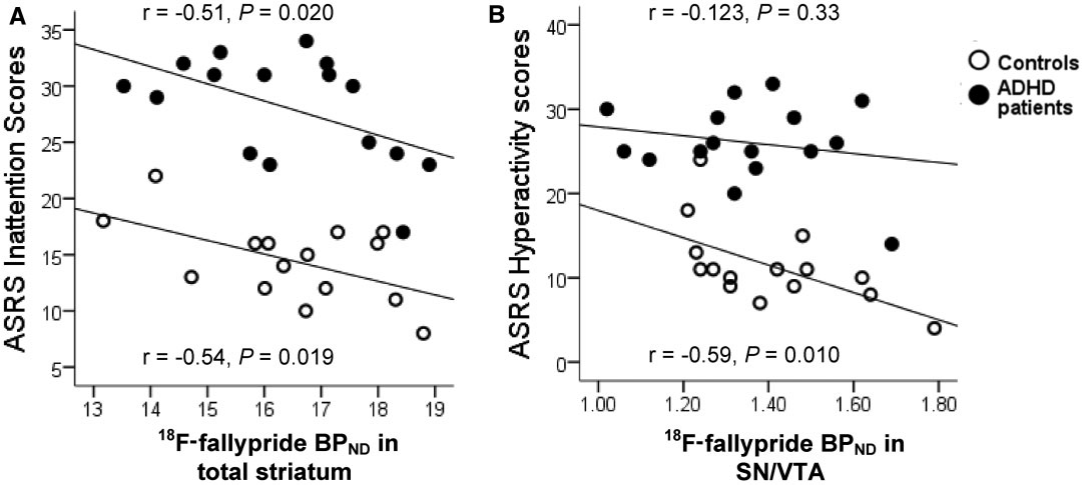
Linear regressions and corresponding Pearson correlations between (**A**) adult ADHD self-rating scale (ASRS) inattention scores and BP_ND_ in total striatum and (**B**) ADHD self-rating scale hyperactivity scores and BP_ND_ in the substantia nigra/ventral tegmental area (SN/VTA) in patients with ADHD and control subjects.

ADHD self-rating scale total and inattention sub-scores correlated negatively with total striatum BP_ND_ both in patients (r* = *−0.48, *P = *0.032; r* = *−0.51, *P = *0.020) and control subjects (r* = *−0.47, *P = *0.04; r* = *−0.54, *P = *0.019) (Fig. 5A) but did not correlate with BP_ND_ in substantia nigra/ventral tegmental area for either subject group. Conversely, self-rated hyperactivity was not associated with BP_ND_ in total striatum but was negatively correlated with BP_ND_ in substantia nigra/ventral tegmental area in control subjects (r* = *−0.59, *P = *0.010) although not in patients (r* = *−0.123, *P = *0.33) (Fig. 5B).

Correlation coefficients for left and right striatal subregions and substantia nigra/ventral tegmental area were similar in patients and control subjects (*P > *0.1). For patients and control subjects combined, inattention scores were negatively correlated with BP_ND_ in the portions of the associative striatum corresponding to pre-commissural dorsal caudate and post-commissural caudate (r* = *−0.393, *P = *0.014; r* = *−0.375, *P = *0.019) and in sensorimotor striatum (r* = *−0.324, *P = *0.038). Hyperactivity scores for all subjects on the other hand were associated with BP_ND_ in left substantia nigra/ventral tegmental area (r* = *−0.34, *P = *0.032) but not in any other region.

#### Relationship between sustained attention (A’) and regional BP_ND_ on placebo

In patients, low A’ scores were associated with low BP_ND_ in left pre-commissural dorsal caudate (r* = *0.48, *P = *0.031) and right post-commissural caudate (r* = *0.43, *P = *0.048). Although the correlation coefficients in control subjects were not significant, they were in the same direction as those observed in patients for all regions and were not significantly different from them (*P > *0.1). Across the entire subject group, low A’ scores were associated with low BP_ND_ in left pre-commissural dorsal caudate (r* = *0.314, *P = *0.04) as well as in left and right post-commissural caudate (r* = *0.308, *P = *0.043 and r* = *0.323, *P = *0.036, respectively; mean: r* = *0.33, *P = *0.032).

Stepwise linear regression of all regional BP_ND_ values on attentional performance (in all subjects) revealed a significant best-fit model with the left pre-commissural dorsal caudate as independent variable [*β = *0.386, *P = *0.029; *F*(1,30)* = *5.26, adjusted R^2^* = *0.21].

### Methylphenidate results

#### Plasma level analysis and cardiovascular effects of methylphenidate

Plasma level analysis of methylphenidate confirmed randomization and compliance with discontinuation of therapeutic medication. Methylphenidate produced significant increases in systolic blood pressure [mean paired difference 5 mmHg; t(31)* = *4.5; *P < *0.001] and heart rate [mean paired difference 7 beats per min; t(31)* = *4.4; *P < *0.001] (Supplementary material). Mean methylphenidate plasma concentrations were similar in patients with ADHD and control subjects (*P > *0.05).

#### Effects of methylphenidate on sustained attention

**Figure 6:**
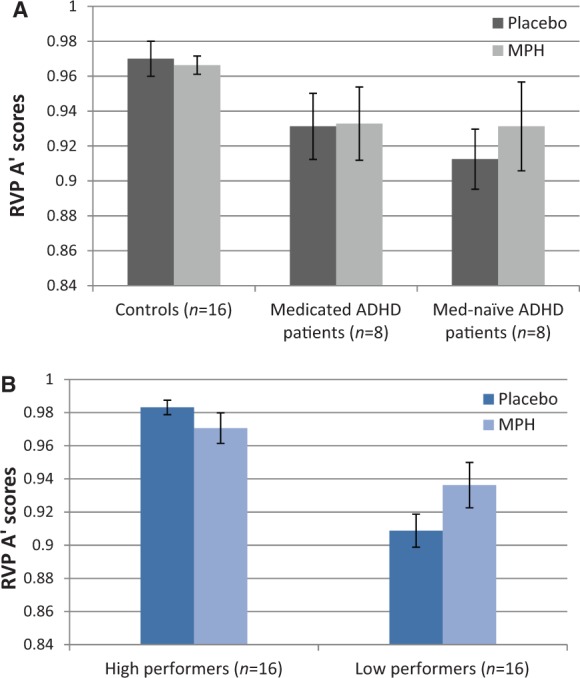
RVP A’ scores following placebo and methylphenidate (MPH) as a function of (**A**) ADHD diagnosis, with the ADHD group split by medication status and (**B**) baseline performance, with the combined ADHD and control groups split by the median of A’.

There was no significant main effect of methylphenidate on A’, nor was there an interaction of methylphenidate with group. Covarying for medication status within the patient groups did not change these results (interactions between treatment and group versus medication status all *P > *0.05) (Fig. 6A).

Based on accumulating evidence that effects of methylphenidate and related drugs on cognition are baseline performance-dependent (Kimberg *et al.*, 1997; Mattay *et al.*, 2000; Mehta *et al.*, 2000; Gibbs and D’Esposito, 2005; Tipper *et al.*, 2005; Finke *et al.*, 2010), changes in sustained attention following methylphenidate were assessed as a function of baseline A’ scores, independent of group. We thus split participants into high and low performers based on the median for A’, as used previously (Kimberg *et al.*, 1997). There was an overlap of performance between patients with ADHD and control subjects in about a third of all subjects. High and low performers were matched for age (mean 28 ± 6 years versus 31 ± 7 years; *P = *0.17), IQ (mean 113 ± 8 versus 115 ± 7) and methylphenidate plasma concentrations (mean 10 ± 3.16 μg/l versus 9.87 ± 3.78 μg/l) (furthermore, four of the five patients with ADHD assigned to the high performing group were on prescribed ADHD medication).

This analysis showed that methylphenidate affected cognition differentially in high and low performing subjects [drug by group interaction: *F*(1,28)* = *10.94, *P = *0.003], improving A’ scores in low [t(15)* = *−2.610, *P = *0.02] but not in high performers [t(15)* = *1.656, *P = *0.119] (Fig. 6B).

#### Effects of methylphenidate on regional BP_ND_

**Figure 7:**
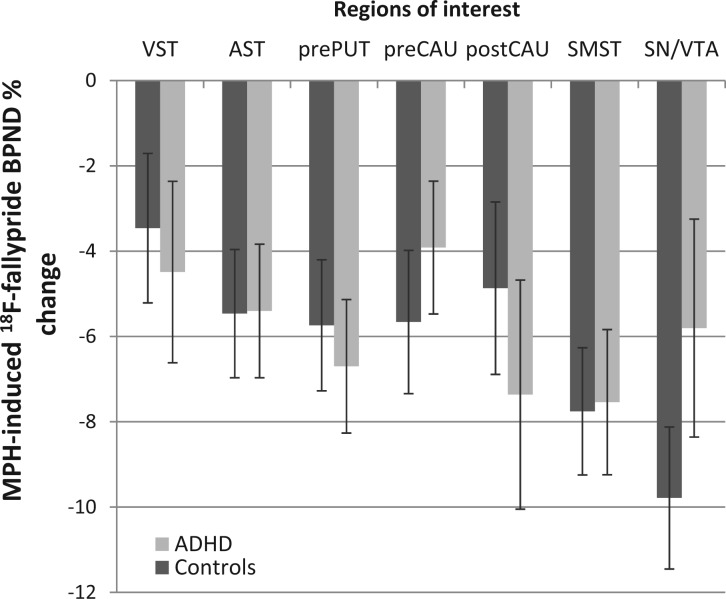
Methylphenidate (MPH)-induced BP_ND_ % change in healthy control subjects and ADHD patients across regions of interest. Reductions in BP_ND_ were significant in all regions and similar in both groups. There was a trend for decreased BP_ND_ % change in substantia nigra/ventral tegmental area in patients with ADHD compared to control subjects [t(30)* = *−1.65, *P = *0.055]. Error bars denote standard error of the mean. VST = ventral striatum; AST = associative striatum; prePUT = pre-commissural putamen; preCAU = pre-commissural caudate; postCAU = post-commissural caudate; SMST = sensorimotor striatum; SN/VTA = substantia nigra/ventral tegmental area.

Methylphenidate significantly reduced BP_ND_ [main effect of treatment: *F*(1,29)* = *30.51, *P < *0.001], ranging from −4.0 to −7.8% depending on anatomical region {region of interest by treatment interaction [*F*(3,87)* = *10.31, *P < *0.001]}, being greatest in substantia nigra/ventral tegmental area and least in ventral striatum (Table 3 and Fig. 7). Methylphenidate also reduced BP_ND_ differentially across ventral striatum, associative striatum and sensorimotor striatum [region of interest by treatment interaction: *F*(2,42)* = *8.34, *P = *0.002], revealing a preferential effect in sensorimotor striatum. The magnitude of regional BP_ND_ % change was similar in patients with ADHD and control subjects (main effect of group and group by drug interaction: *P > *0.05).

#### Baseline performance-dependent relationship between methylphenidate effects on sustained attention (A’ % change) and BP_ND_ % change

Methylphenidate-induced % change in A’ was negatively correlated with BP_ND_ % change in ventral striatum (r* = *−0.29, *P = *0.05) and positively with substantia nigra/ventral tegmental area (r* = *0.5, *P = *0.002), with the correlation coefficients being highly significantly different (z* = *3.25, *P < *0.001).

**Figure 8:**
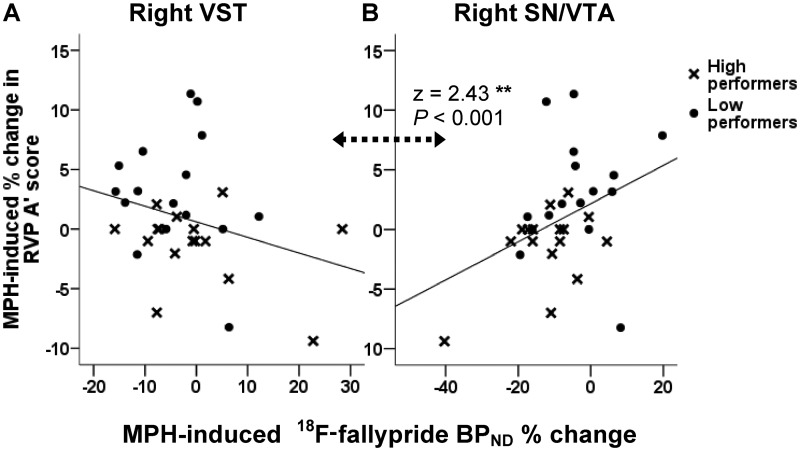
Regression lines representing the relationship between methylphenidate (MPH) effects on RVP A’ scores and ^18^F-fallypride BP_ND_ % change in (**A**) right ventral striatum (VST) and (**B**) right substantia nigra/ventral tegmental area (SN/VTA). Correlation coefficients in these regions were significantly different (z* = *2.43, *P < *0.001). Note the *x*-axis scales are different.

After controlling for between-subject differences in methylphenidate plasma levels, improvements in sustained attention were associated with greater BP_ND_ % change in right ventral striatum (r* = *−0.318, *P = *0.043) and lower BP_ND_ % change in substantia nigra/ventral tegmental area [r* = *0.32, *P = *0.043; r* = *0.309, 8 *P = *0.048; r* = *0.374, *P = *0.021; in left, right and bilateral substantia nigra/ventral tegmental area, respectively (Fig. 8)]. Correlation coefficients for ventral striatum and substantia nigra/ventral tegmental area were significantly different from each other (*P < *0.01), but similar in patients with ADHD and control subjects.

The above analyses were also calculated after excluding ceiling performers on the attentional task (five control subjects and one patient), yielding similar results (Supplementary material).

We hypothesized that the relationship between A’ % change and BP_ND_ % change for both ventral striatum and substantia nigra/ventral tegmental area would be dependent on A’ baseline scores, based on the aforementioned differential effect of methylphenidate in high and low performers. Controlling for between-subject differences in A’ scores (in addition to methylphenidate plasma levels) had no impact on the correlation between methylphenidate-induced changes in A’ scores and BP_ND_ % change in ventral striatum (r* = *−0.348, *P = *0.032 for bilateral ventral striatum; r* = *−0.247, *P = *0.098 and r* = *−0.349, *P = *0.033 for left and right ventral striatum, respectively). Therefore, these effects were not baseline-dependent.

**Figure 9:**
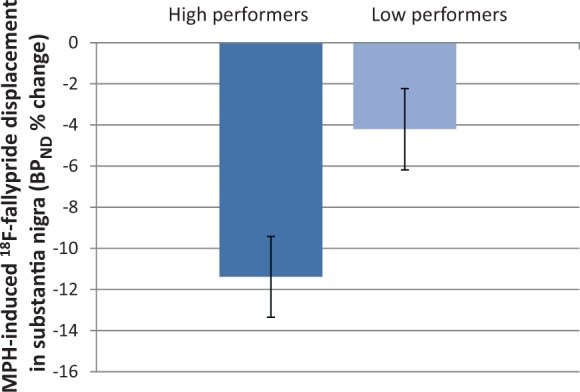
BP_ND_ % change in substantia nigra/ventral tegmental area for high and low performers. Low performers had decreased BP_ND_ % change following methylphenidate (MPH) (*P = *0.007), suggesting that drug-induced increase in endogenous dopamine in this region was smaller than for high performers.

On the contrary, changes in sustained attention associated with BP_ND_ % change in substantia nigra/ventral tegmental area were dependent on baseline A’ scores, the correlations for this region no longer being significant (r* = *0.223, *P = *0.142 for bilateral substantia nigra/ventral tegmental area; r* = *0.144, *P = *0.228 and r* = *0.218, *P = *0.129 for left and right substantia nigra/ventral tegmental area, respectively). Corroborating this finding, repeated measures ANOVA revealed lower BP_ND_ % change in low compared with high performers in substantia nigra/ventral tegmental area (Fig. 9) [*F*(1,28)* = *9.351, *P = *0.005; *F*(1,28)* = *5.46, *P = *0.027 and *F*(1,28)* = *5.2, *P = *0.030 for bilateral, left and right substantia nigra/ventral tegmental area, respectively], but not in any other region.

Stepwise linear regression modelling to determine the relative impact of methylphenidate-induced BP_ND_ changes across regions and methylphenidate plasma levels on drug-induced A’ changes confirmed the role of the right midbrain in predicting changes in attentional performance [*β = *0.39, *P = *0.30; *F*(1,30)* = *5.21, adjusted R^2^* = *0.12]. A further regression model revealed that baseline performance levels on A’ predicted BP_ND_ changes specifically in the left midbrain [*β = *−0.471, *P < *0.01; *F*(1,30)* = *8.57, adjusted R^2^* = *0.20], confirming the above correlational results.

#### Baseline performance-dependent regional BP_ND_

**Figure 10:**
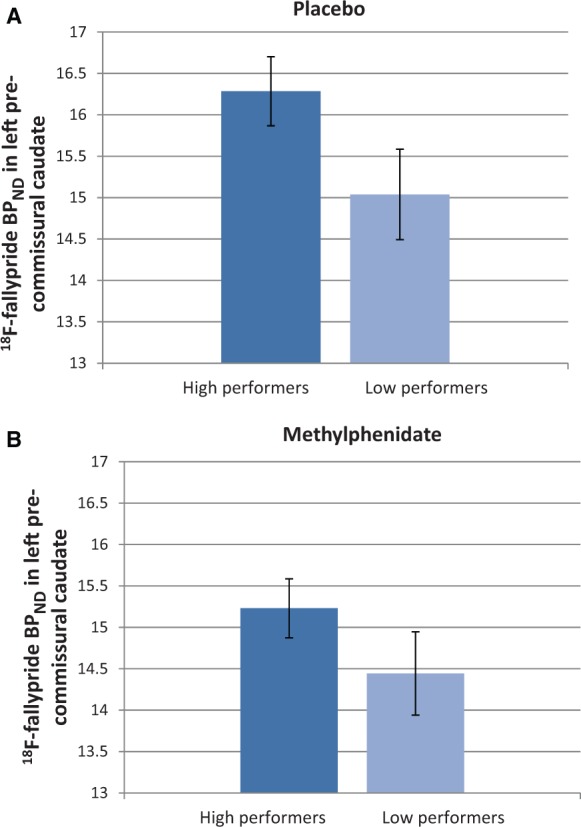
BP_ND_ in left pre-commissural caudate in high and low performers following (**A**) placebo and (**B**) methylphenidate (MPH). Low performers had reduced BP_ND_ in left pre-commissural caudate on placebo (*P = *0.035), which was normalized by methylphenidate.

On placebo, low performers had reduced BP_ND_ in left pre-commissural dorsal caudate (Fig. 10A) [*F*(1,28)* = *4.63, *P = *0.04]. As shown in Fig. 10B, on methylphenidate this group difference was no longer observed [*F*(1,28)* = *2.97, *P = *0.096].

## Discussion

This study examined the neural basis of the attentional deficits associated with ADHD and the mechanisms underpinning a single therapeutic dose of methylphenidate through the application of multi-modal state-of-the-art neuroimaging techniques. Adult patients with ADHD as a group had marked deficits in sustained attention and showed reduced grey matter volume in distributed frontostriatal and limbic circuits relative to control subjects. No significant group differences in D_2_/D_3_ receptor availability were found; however, poor performance on the computerized sustained attention task across both patient and control groups was associated with low BP_ND_ values in the left caudate. Methylphenidate increased dopamine levels across all striatal regions as well as the midbrain similarly in patients with ADHD and control subjects, thereby normalizing D_2_/D_3_ receptor availability in low performers. The drug improved sustained attention in a baseline-dependent manner independently from diagnosis. Subjects performing poorly at baseline also had significantly smaller drug-induced dopamine release in the midbrain. Collectively these novel results suggest that deficits in sustained attention are associated with both reduced D_2_/D_3_ receptor availability in the left caudate on placebo and reduced dopamine release in the midbrain in response to methylphenidate, and that these relationships hold irrespective of ADHD diagnosis.

### Neural correlates of ADHD

The absence of overall group differences in ^18^F-fallypride binding in this study may seem to be at variance with previous reports of reduced ^11^C-raclopride binding in selected brain regions of the left hemisphere in adult patients with ADHD compared with control subjects (Volkow *et al.*, 2007*b*, 2009). However, it is possible that the apparent discrepancy can be resolved by the following consideration: low performing subjects drawn from both ADHD and control groups on the computerized test of sustained attention in fact had significantly lower BP_ND_ (attributed to lower D_2_/D_3_ receptor availability) in the left pre-commissural caudate than high-performing subjects, though independent of diagnosis. This result is consistent with previous evidence implicating the left caudate in inattentiveness, not only in patients with ADHD, but also in healthy subjects (Schrimsher *et al.*, 2002; Volkow *et al.*, 2007*a*, *b*, 2009) and is also congruent with reports that lesions in the left caudate produce attentional deficits (Benke *et al.*, 2003).

Our findings define a role for D_2_/D_3_ receptor availability in the manifestation of attentional deficits that can be explained in the light of the previously hypothesized continuum model of cognitive deficits and underlying dopaminergic dysregulation associated with ADHD (Sahakian and Robbins, 1977). Consistent with the model, the attentional deficits observed in ADHD fall along a single normal continuum extending into the healthy population, with patients with ADHD predominantly representing the lower end of the distribution. However, the lack of overall group differences in ^18^F-fallypride binding argue against a primary nigro-striatal-dependent dopamine dysfunction in adult ADHD. Thus, although our data suggest that dopamine plays a key role in attention deficits, they also imply that dopaminergic deficits alone do not account for an ADHD diagnosis. What is then different in ADHD in neural terms?

Of course it is possible that such differences in dopamine function may exist within other regions not addressed here such as the frontal cortex (e.g. Ernst *et al.*, 1998, but see also Del Campo *et al.*, 2011*a* for discussion). However, the sensitivity of ^18^F-fallypride to the methylphenidate dose administered in the current design was arguably suboptimal for detecting robust changes in dopamine levels in regions with low D_2_/D_3_ receptor density such as the prefrontal cortex. A further possibility is that the central noradrenergic system is selectively affected in ADHD. In support of this hypothesis, atomoxetine, a selective noradrenergic reuptake inhibitor, has been reported to be effective in the treatment of ADHD and some of its associated cognitive deficits (Chamberlain *et al.*, 2007). Finally, it may also be argued that ADHD as described in the DSM-IV encompasses an array of phenotypes and underlying mechanisms too broad and heterogeneous to be associated with a primary neurobiological dysfunction common to all patients. The Research Domain Criteria project (RDoC) recently launched by the National Institute of Mental Health, which encourages the development of new ways of classifying psychopathology based on dimensions of observable behaviour and neurobiological measures, represents an important step forward for future research which may help encapsulate the heterogeneity in ADHD. However, it is striking that although no group differences were found in terms of dopamine activity in our study, the same group of adult patients with ADHD had significant brain tissue abnormalities in frontostriatal and limbic structures compared with control subjects.

### Methylphenidate-induced dopamine increases: regional dependence and therapeutic implications

Some of the present findings on effects of methylphenidate are relevant to the more general understanding of stimulant drug action in healthy humans, other than simply in patients with ADHD. Thus, here we report, for the first time, that therapeutic doses of methylphenidate administered orally reduce ^18^F-fallypride binding, which can be attributed to methylphenidate-induced increases in synaptic dopamine levels. Effect sizes and rank order of ^18^F-fallypride BP_ND_ % change across subregions of the striatum and the midbrain were comparable with those documented following oral administration of amphetamine (Riccardi *et al.*, 2006*a*; Cropley *et al.*, 2008) (Supplementary material), the alternative first-line treatment for ADHD which also increases catecholamine levels, albeit via different cellular mechanisms (Faraone and Glatt, 2010). Consistent with previous reports, the substantia nigra/ventral tegmental area showed high levels of BP_ND_ % change, which were comparable to those observed in sensorimotor striatum.

These findings replicate the preferential effect of methylphenidate in the sensorimotor striatum when administered orally using ^11^C-raclopride binding (Clatworthy *et al.*, 2009). The preferential effect of oral methylphenidate and amphetamine on dorsal compared to ventral regions of the striatum, however, is in contrast with the well-replicated finding that intravenous amphetamine leads to greater dopamine changes in ventral compared to dorsal striatum (Drevets *et al.*, 1999, 2001; Martinez *et al.*, 2003). Animal evidence suggests that the ability to increase dopamine in ventral striatum, involved with reward circuitry, is a common pharmacological effect underlying the reinforcing properties of virtually all drugs of abuse (Di Chiara and Imperato, 1988; Bradberry *et al.*, 2000). Consistent with these data, in healthy humans, amphetamine-induced dopamine changes in ventral but not dorsal striatum were found to predict hedonic responses (Drevets *et al.*, 2001; Leyton *et al.*, 2002; Martinez *et al.*, 2003).

The pattern of dopamine changes observed along the ventral–dorsal gradient of the striatum following oral versus intravenous administration of methylphenidate and amphetamine may be explained in light of the different pharmacokinetics associated with each route of drug administration. Whereas oral methylphenidate penetrates the brain only slowly, peaking 60–90 min after administration, intravenous methylphenidate rapidly enters the brain, peaking in <15 min. The large and abrupt dopamine increase after intravenous administration is perceived as reinforcing, an effect that appears to be reduced when the increase in dopamine is slow and progressive (Volkow, 2006).

Overall, the differences in regional patterns of methylphenidate-induced dopamine increase following oral versus intravenous methylphenidate administration suggest that the mechanisms underlying the effects of the drug to improve attention differ from those mediating its reinforcing actions.

### Baseline performance-dependent effects of methylphenidate

Methylphenidate significantly improved sustained attention in a baseline-dependent manner in low performers from both groups. Although the lack of methylphenidate effects on attention in high performers was possibly due to ceiling effects, this does not undermine the key finding that the drug is capable of improving sustained attention in low-performing individuals, regardless of ADHD diagnosis. This result adds to previous evidence demonstrating equivalence of behavioural stimulant effects in individuals with and without ADHD (Rapoport *et al.*, 1978, 1980), supporting a dimensional model of attentional deficits and underlying catecholaminergic mechanisms of ADHD which extends into the healthy population. It is also consistent with a growing literature documenting baseline performance-dependent effects of stimulants and related drugs, which have been explained by a hypothesized inverted U-shaped function, whereby optimal catecholamine levels determine optimal performance, and levels along the curve at either side of the optimum are associated with impaired performance (Robbins and Sahakian, 1979; Naylor *et al.*, 1985; Williams and Goldman-Rakic, 1995; Kimberg *et al.*, 1997; Arnsten and Goldman-Rakic, 1998; Rogers *et al.*, 1999; Mattay *et al.*, 2000, 2003; Tipper *et al.*, 2005; Finke *et al.*, 2010). Inverted U-shaped functions as invoked here accurately describe the dose-response relationships often observed in psychopharmacological studies of cognition (Dews, 1958; Sahakian and Robbins, 1977; Cools and Robbins, 2004; Robbins, 2010). Our data do not allow drawing conclusions on the declining portion of the hypothesized function. Further research is needed to test the full dose-response curve of methylphenidate, which would require higher doses than the one used here.

**Figure 11:**
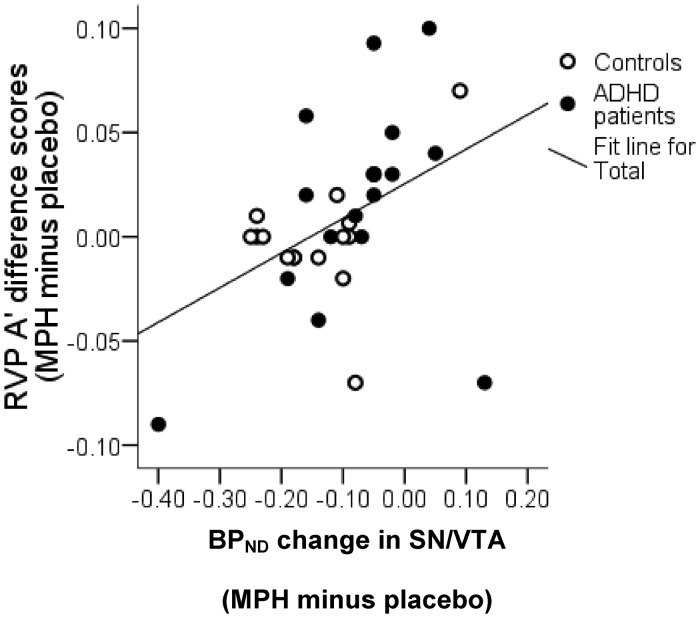
Regression line representing the relationship between methylphenidate (MPH) effects on sustained attention and on ^18^F-fallypride BP_ND_ in the midbrain (r* = *0.502; *P = *0.002, two-tailed). SN/VTA = substantia nigra/ventral tegmental area.

A central finding of this study was that the drug-induced changes in performance correlated with changes in midbrain dopamine levels (Fig. 11), a result that remained unchanged after excluding ceiling performers. Changes in midbrain dopamine levels mirrored the behavioural baseline-dependent effect: low performers had reduced methylphenidate-induced BP_ND_ reductions in the midbrain compared with high performers, with smaller dopamine changes being associated with greater improvements on the sustained attention task. These robust neurobiological correlates argue against the behavioural baseline-dependent effects resulting simply from a statistical regression towards the mean.

These findings suggest that the effects of methylphenidate on midbrain dopamine levels play a hitherto unknown role in the modulation of attention. A further likely mechanism by which methylphenidate improved sustained attention in low performers is the observed normalization of dopamine levels in the left caudate.

### Midbrain mechanisms underlying therapeutic effects of methylphenidate

**Figure 12:**
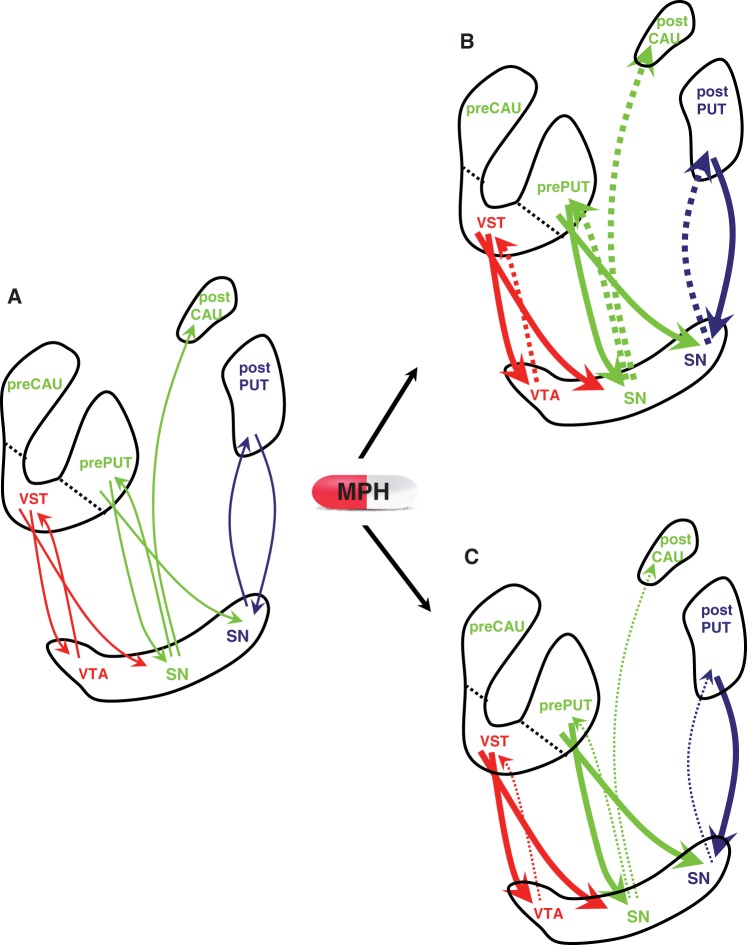
Schematic model of dopamine activity along striato-nigro-striatal circuits at baseline (**A**) and after oral methylphenidate (MPH) in high (**B**) and low performers (**C**). Ascending arrows represent projections from dopamine (DA) neurons located in the substantia nigra (SN)/ventral tegmental area (VTA) to the striatum and descending arrows represent GABA-ergic projections from the striatum to the substantia nigra/ventral tegmental area. Different colours represent different striatal functional circuits (red* = *limbic, green* = *associative, blue* = *sensorimotor). Methylphenidate administration (**B**) increased postsynaptic catecholamine stimulation (solid lines), leading to inhibition of dopamine neuron firing (dashed lines) by activation of synthesis- and release-regulating dopamine autoreceptors. It is suggested that the level of responsivity of dopamine neurons and subsequent striato-nigro-striatal neuromodulation following methylphenidate administration was dependent on baseline dopamine activity in the striatum. Adapted from Haber *et al.* (2000) and Martinez *et al.* (2003). CAU = caudate PUT = putamen; VST = ventral striatum.

Methylphenidate-induced dopamine changes in substantia nigra/ventral tegmental area were negatively related to dopamine changes in ventral striatum but not associative striatum or sensorimotor striatum. These results may reflect regional differences in the response of midbrain dopamine neurons following methylphenidate administration. Dopamine neurons located in the midbrain receive γ-aminobutyric acid (GABA)-ergic projections from the same striatal subregion they project to through reciprocal connections, giving rise to topographically organized striato-nigro-striatal circuits (Haber *et al.*, 2000). Additionally, this system provides non-reciprocal connections, facilitating dopamine transmission along a feed–forward spiral starting in the ventral striatum and finishing in the sensorimotor striatum, whose projections to the midbrain are confined to the regions from which it receives its dopamine input (Haber *et al.*, 2000) (Fig. 12A). GABAergic neurons descending from the striatum to the midbrain can inhibit or stimulate dopamine cell firing depending on whether they contact dopamine neurons or GABAergic interneurons. After administration of psychostimulants (Bunney and Achajanian, 1976), including methylphenidate (Shi *et al.*, 2004), the inhibitory effect prevails, leading to decreased responsiveness in dopaminergic neurons (Fig. 12B).

We hypothesize that the inverse relationship in dopamine release between substantia nigra/ventral tegmental area and ventral striatum but not associative striatum or sensorimotor striatum following methylphenidate reflects the asymmetrical nigro-striatal projection pattern. Ventral striatum is the only striatal region whose dopamine input is unaffected by non-reciprocal connections, its dopaminergic input thus being regulated to a greater extent by synthesis- and release-regulating midbrain dopamine autoreceptors. The observed inverse correlation may thus reflect the inhibitory influences exerted by midbrain dopamine neurons to regulate dopamine levels in ventral striatum. This inverse relationship did not hold for associative striatum and sensorimotor striatum, which can be explained by the fact that, via the aforementioned feed–forward spiralling connections, associative striatum receives additional inhibitory influences from ventral striatum and sensorimotor striatum from both ventral striatum and associative striatum. These findings possibly constitute the first direct evidence in humans supporting the notion that the doses used clinically to treat ADHD exert their therapeutic actions through presynaptic mechanisms. A key finding of this study is that midbrain dopamine autoreceptor regulation was reduced in low performers, as evidenced by the significantly smaller methylphenidate-induced increases in midbrain dopamine levels observed in low compared to high performers (Fig. 12C).

How are the changes in midbrain dopamine receptor availability related to the attention-enhancing effects of methylphenidate, whether in patients with ADHD or healthy control subjects? A plausible explanation is provided by the classical model of catecholamine neurotransmission regulation and psychostimulant action postulated by Grace (1991, 2001). According to this model, the amplitude of the phasic dopamine response is dynamically regulated by the influence of corticostriatal activity through the modulation of tonic dopamine levels. Low tonic dopamine activity is associated with abnormally high phasic dopamine responses, resulting in distractibility and impaired attention. Stimulants enhance tonic catecholamine levels, thereby increasing postsynaptic catecholamine receptor stimulation but also triggering negative feedback mechanisms through stimulation of dopamine autoreceptors. As a result, dopamine synthesis is reduced, dopamine neuron firing inhibited and subsequent phasic (spike-dependent) transmitter release reduced.

The idea that the clinical effects of stimulants are mediated by the ability of small increases in extracellular dopamine to produce a net decrease in phasic catecholamine release by the selective activation of dopamine autoreceptors (following an increase in tonic dopamine levels) (Solanto, 1984, 1998; Seeman and Madras, 1998) is supported by studies showing reduction in behavioural activity levels in children with ADHD following low doses of dopamine agonists in the range presumed to stimulate autoreceptors (Solanto, 1986) and from neurocomputational models (Oades *et al.*, 1985; Sawaguchi *et al.*, 1988; Daniel *et al.*, 1991; Servan-Schreiber *et al.*, 1998; Li *et al.*, 2001; MacDonald *et al.*, 2009). It can thus be concluded that stimulant-induced increases in endogenous midbrain dopamine in the presence of reduced tonic striatal dopamine levels observed in low performers may improve the signal-to-noise ratio, thereby normalizing the system to approximate that characterizing high performing subjects.

### Strengths and limitations

In an attempt to identify putative biomarkers of ADHD, we used, for the first time, an interdisciplinary approach encompassing both PET and MRI in combination with neuropsychopharmacological assessment which allowed characterization of the disorder at clinical, cognitive, neurochemical and neuroanatomical levels. The current paper focused on the neuropsychopharmacological findings; a companion paper will address in more detail grey and white matter tissue in relation to the behavioural changes.

An important innovation of this study relative to previous ADHD PET studies was the use of high resolution co-registered MRI images to help localize the PET signal. This was crucial to allow segmentation of the striatum into its functional subregions, known to have different tracer uptake due to heterogeneous D_2_/D_3_ receptor density (Del Campo *et al.*, 2011*b*).

A further strength was the experimental design, (double-blind, randomized, cross-over) which differs from the fixed design (placebo first, followed by drug) used in previous studies investigating the effects of methylphenidate in ADHD (Rosa-Neto *et al.*, 2005; Volkow *et al.*, 2007*b*). It might be argued that placebo is not equivalent to a null condition, and thus the comparison between studies using placebo as a control condition with those in which baseline measurements are acquired might be suboptimal. Indeed, PET evidence has shown that sensitization to amphetamine in humans leads to carry-over effects in subsequent sessions when placebo is administered (Boileau *et al.*, 2007). These findings imply that placebo administered in the session subsequent to stimulant treatment can potentially reduce the power to detect true stimulant effects and identify neurobiological markers in psychiatric populations. However, fixed designs entail order effects, thereby introducing the confounding effect of drug anticipation. PET evidence suggests that anticipation of stimulants such as caffeine can induce dopaminergic responses in humans (Kaasinen *et al.*, 2004). To address this issue, placebo ^18^F-fallypride BP_ND_ values were compared with ^18^F-fallypride BP_ND_ estimates documented by other groups in the same regions of interest at baseline in fixed designs (Riccardi *et al.*, 2006*a*; Cropley *et al.*, 2008; Slifstein *et al.*, 2010) (Supplementary material). Values were largely comparable in terms of both magnitude and rank order, suggesting that the regional BP_ND_ values reported here following placebo administration were within the expected baseline ranges. One way future research might help to disentangle undesired effects of drug-order and stimulant carry-over effects is through implementation of a double-blind randomized design with parallel groups, as used by Aalto *et al.* (2009) where each subject underwent two scans; the first at baseline and the second after administration of either a psychostimulant or placebo. However, there is no evidence to suggest that our design produced anomalous results.

A caveat of dopamine receptor imaging technique as the one reported here is that receptor availability and dopamine release are inferred from estimates of BP_ND_, which is a combined measure of receptor availability and radioligand affinity. Consequently, we were not able to assess whether dynamics such as receptor internalization (Laruelle, 2000) or changes in the affinity of D_2_/D_3_ receptors for dopamine and/or fallypride were implicated (Laruelle, 2000; Gjedde *et al.*, 2005). In the absence of information regarding levels of endogenous dopamine, it was not possible to determine to what extent D_2_/D_3_ receptor availability reflected receptor density or basal occupancy. Moreover, this technique did not allow a distinction of the proportion of different D_2_/D_3_ receptor types (synaptic versus extra-synaptic, post versus presynaptic) or their state of affinity for dopamine. It is important to note also that the slow kinetics of ^18^F-fallypride necessitated a long acquisition period (3.15 h) and thus did not allow us to differentiate tonic from phasic dopamine activity in the midbrain, which can only be inferred.

A further methodological aspect that merits discussion concerns partial volume effects, defined as the loss in spatial resolution resulting in spill-over of image signal to and from adjacent structures. Specifically, partial volume effects are to be considered as a potential confounding factor of radiotracer uptake when the structures under study are small and/or when adjacent structures have high signal contrast. ^18^F-fallypride PET imaging of the striatum satisfies both these criteria and hence partial volume effects are theoretically an issue of concern in this study. Moreover, our finding of reduced grey matter volume in the putamen in patients with ADHD compared with control subjects implies that partial volume effects may have had a differential effect on sub-striatal BP_ND_ estimation in patients and control subjects, potentially masking group differences in regional D_2_/D_3_ receptor availability. In order to rule this out, an additional set of analyses was carried out to estimate partial volume error using an in-house implementation of a well-validated method (Rousset *et al.*, 1998). A detailed account of the methodology used and the results can be found in the Supplementary material. Importantly, signal recovery due to partial volume effect was similar in patients with ADHD and control subjects, independently from brain region and experimental condition, and thus it can be concluded that the grey matter changes observed in patients did not confound the PET results here reported.

Finally, the medication history of the patients also needs discussing, as half of the patients in the current study had been previously exposed to ADHD medication. Although this study was not designed to resolve the question of whether patients with ADHD with a history of pharmacological treatment have different D_2_/D_3_ receptor availability compared to medication-naïve patients, we did not find any differences in PET measurements between patients with previous exposure to ADHD medication and medication-naïve patients (all *P > *0.05). Importantly, to ensure complete washout at the time of scanning among patients under current treatment, compliance with treatment discontinuation 3 days before each PET scan was verified through urine screening. Moreover, medication status was controlled for in all repeated measures ANOVAs. There is a lack of clarity regarding chronic effects of ADHD medication, with different studies reporting contradicting results with regard to its impact on dopaminergic markers (Gill *et al.*, 2012; Volkow *et al.*, 2012). Further research and suitable study designs are needed to fully characterize the consequences of long-term pharmacological ADHD treatment both at the behavioural and at the neurobiological levels.

## Conclusion

The present findings query the precise role of dopamine in the pathophysiology in adult ADHD. Although our findings are consistent with the modulation of attention by nigro-striatal dopamine and with poor attention being a key deficit in the clinical profile of ADHD, our data also suggest that dopamine dysregulation *per se* is unlikely to be the primary cause underlying ADHD pathology in adults. This conclusion is reinforced by evidence of structural brain changes in the same set of patients with adult ADHD.

We also conclude that a more precise explanation of the behavioural effects of psychostimulant treatment in both patients with ADHD and healthy control subjects can be derived from the combination of (i) dose and time course of methylphenidate actions; (ii) level of responsivity of the dopamine system determined by tonic dopamine levels; and (iii) baseline level of performance, possibly reflecting basal dopamine function in the striatum. The findings of reduced methylphenidate-induced midbrain dopamine increases and improved sustained attention in low performers (mostly patients with ADHD) implicate a hitherto neglected role of midbrain dopamine in the mediation of therapeutic effects of methylphenidate.

We have shown the considerable potential of combining the complementary strengths of structural MRI and neuroreceptor PET imaging in identifying and characterizing cognitive endophenotypes of ADHD, which may help our understanding of the diagnosis, prognosis and treatment of ADHD.

## Supplementary Material

Supplementary Data

Supplementary Data

## Abbreviations

AC–PC: anterior commissure–posterior commissure
ADHD: attention deficit/hyperactivity disorder
BP_ND_: ^18^F-fallypride binding potential
DSM: Diagnostic and Statistical Manual of Mental Disorders
MR_AC-PC_: scans manually aligned to the anterior commissure-posterior commissure line
RVP: Rapid Visual Information Processing
