# Supplementary material for: A positron emission tomography study of nigro-striatal dopaminergic mechanisms underlying attention: implications for ADHD and its treatment
Source: Brain. 2013 Oct 25;136(11):3252–70. doi: 10.1093/brain/awt263 (PMC4125626; doi:10.1093/brain/awt263)
Supplement: Supplementary Data [file supp_136_11_3252__index.html]

Supplementary Data 

# A positron emission tomography study of nigro-striatal dopaminergic mechanisms underlying attention: implications for ADHD and its treatment

## Supplementary Data

files

**Files in this Data Supplement:**

- Supplementary Data - doc file
