# Supplementary material for: A positron emission tomography study of nigro-striatal dopaminergic mechanisms underlying attention: implications for ADHD and its treatment
Source: Brain. 2013 Oct 25;136(11):3252–70. doi: 10.1093/brain/awt263 (PMC4125626; doi:10.1093/brain/awt263)
Supplement: Supplementary Data [file supp_awt263_brain-2012-02105-File015.doc]

**Online supplementary material**

**S.1: Plasma MPH quantification**

Determination of MPH in plasma samples was performed by liquid chromatography/electrospray ionization mass spectrometry . After solid-phase extraction of plasma samples on Waters Oasis HLB cartridges, chromatographic separation was achieved by use of an Ultrasep RP18E column and deuterated MPH as internal standard. The limit of quantitation was 2 µg/L.

**S.2:** **Voxel-wise analysis of [^18^F]fallypride BP_ND_ maps**

Parametric mapping of binding potential BP_ND_ maps were calculated using an in-house Matlab (The MathWorks Inc., Natick, MA) implementation of the simplified reference tissue model [sRTM; ] solved using basis functions . Whole-brain group differences in BP_ND_ on the placebo BP_ND_ maps were investigated using CAMBA.

Baseline [^18^F]fallypride BP_ND_

No group differences were observed between ADHD patients. Voxel-wise linear regression between [^18^F]fallypride BP_ND_ and A’ scores revealed a positive correlation between A’ and BP_ND_ in left caudate and putamen when ceiling performers were excluded (n=6, of which n=5 were healthy controls).


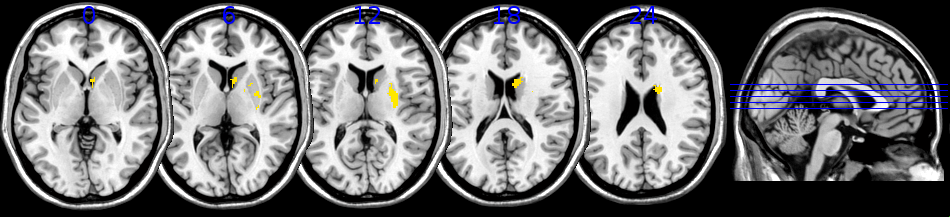

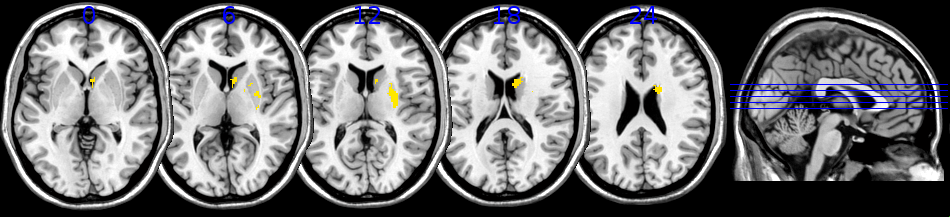


Figure 1: Cluster showing a positive association between A’ scores and placebo BP_ND_ in caudate and putamen in the left hemisphere (p<0.01, corrected for family-wise errors)

MPH-induced [^18^F]fallypride BP_ND_ change

In order to investigate the effects of MPH on BP_ND_ across all voxels, a 2 by 2 repeated measures factorial analysis with group as between subject factor and treatment as a within subject factor was carried out. No significant group differences were found. MPH had a robust effect in striatum and midbrain (replicating the regional results presented in the results section), but also in globus pallidus, limbic regions (hippocampus and amygdala) and temporal cortices (Figure 2). We did not detect MPH effects in cortical regions such as the dorsolateral, orbitofrontal, or medial frontal cortex.


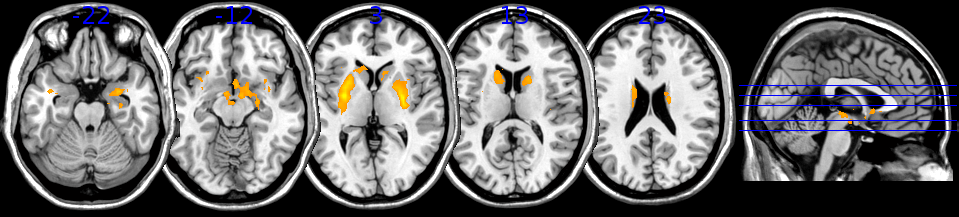

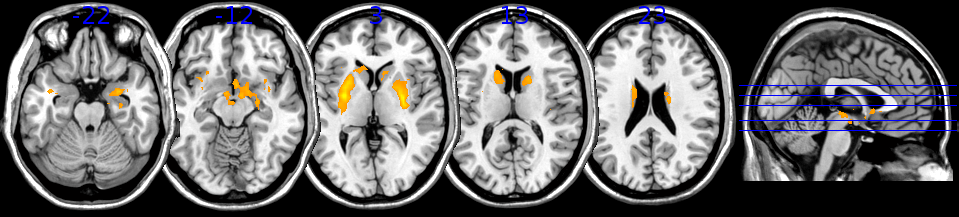


Figure 2: MPH increased endogenous DA levels not only in striatum and midbrain, but also in globus pallidus, limbic regions (hippocampus and amygdala) and temporal cortices (p<0.01, corrected for family-wise errors).

While the existing D_2_/D_3_ receptor imaging literature largely converges with regard to stimulant-induced changes in tracer binding in the striatum and midbrain, results regarding cortical effects have been less consistent. Using [^11^C]FLB 457, Montgomery et al (2007) reported significant extracellular DA increases following oral MPH in frontal and temporal cortices, anterior cingulate, amygdala, hippocampus and thalamus. More recently, Volkow et al reported DA increases following i.v. MPH also in frontal and temporal regions using [^11^C]raclopride (2012). In their 2006 publication reporting effects of oral AMPH on [^18^F]fallypride binding, Riccardi et al. reported displacement of 3-4 % in temporal cortex (no other cortical effects were examined). Cropley et al. (2008) subsequently reported effects of oral AMPH of up to 7 % in temporal, medial and orbital prefrontal cortices. These displacements did not remain significant after controlling for the false discovery rate, presumably because of intersubject variability. The magnitude of regional percent change exceeded the test-retest reliability of baseline [^18^F]fallypride measurement, indicating that the observed changes in BP_ND_ were not due to poor reproducibility.

In a subsequent study using i.v. administration of AMPH, Slifstein et al. (2010) did not find significant changes in [^18^F]fallypride BP_ND_ in medial and orbital prefrontal cortices, in line with our own findings. The authors argued that the lack of effects in these areas might be explained by the combination of low receptor density (i.e. low placebo [^18^F]fallypride values) and high variability of MPH-induced BP_ND_ % change measures, which is likely to also apply in the current study.

**S.3: Comparison of [^18^F]fallypride BP_ND_ values with the literature**

Regional [^18^F]fallypride BP_ND_ values reported in the results section of this paper were directly compared to values of prior [^18^F]fallypride PET studies investigating psychostimulant effects on synaptic DA levels across striatal sub-regions and the midbrain . Cropley et al additionally investigated test-retest reliability of baseline [^18^F]fallypride BP_ND_ in 14 healthy subjects, concluding that [^18^F]fallypride provided robust baseline measures . As shown in Figure 3, BP_ND_ values generated from the placebo scans are comparable to baseline values reported by other groups in the same regions, regarding both magnitude and rank order. Slight variations are likely due to differences in ROI delineation.

Figure 3: Comparison of the placebo [^18^F]fallypride BP_ND_ values reported in the results section of this paper with the baseline values reported in Slifstein et al 2010 (Table III) and Cropley et al 2008 (Tables IV and V).


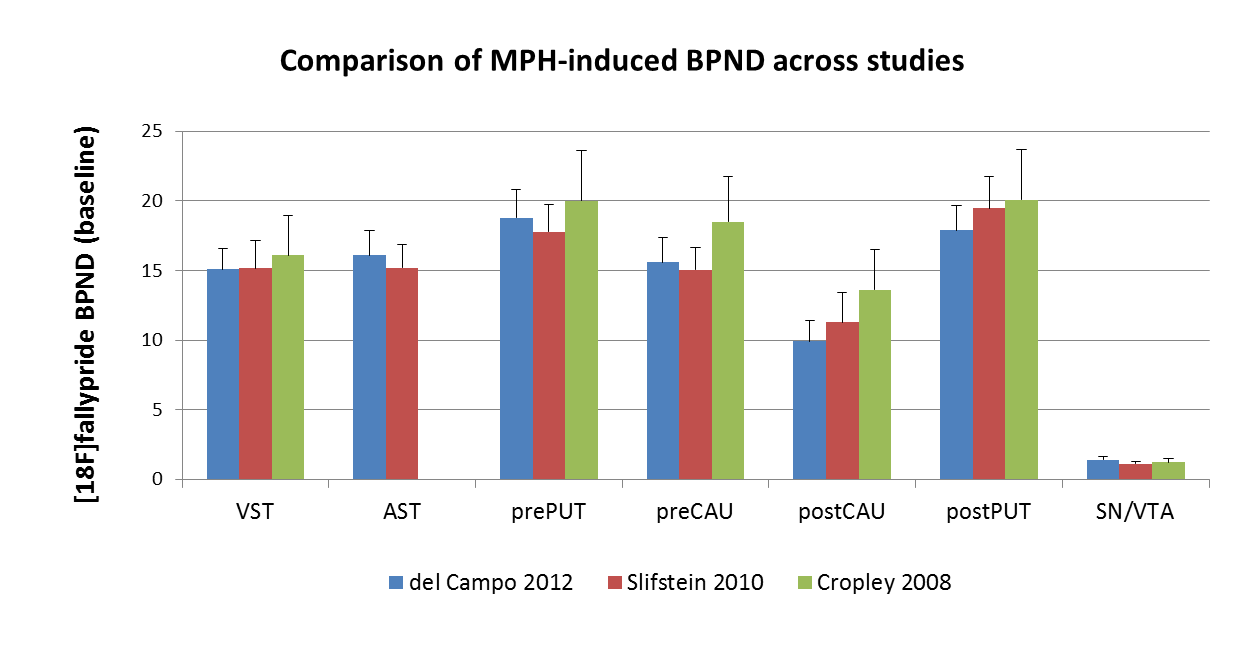


**S.4: Physiological parameters of MPH**

MPH increased systolic blood pressure and heart rate in both groups (Figure 4). The peaks observed at 3 and 5 hours in all cardiovascular measurements coincide with the time points when participants were taken out of the scanner for the break and at the end of the scanning procedure, respectively.

Figure 3: Effects of MPH on cardiovascular parameters in adults with ADHD and controls.

**S.5:** **Correlational analyses without ceiling performers**

MPH-induced % change in A’ was negatively correlated with BP_ND_ % change in VST (r=-0.36, p=0.034) and positively with SN/VTA (r=0.6, p=0.002).After controlling for between-subject differences in MPH plasma levels, improvements in sustained attention were associated with greater BP_ND_ % change in right VST (r=-0.43, p=0.018) and lower BP_ND_ % change in SN/VTA [r=0.380, p=0.033; r=0.31, p=0.070; r=0.40, p=0.025; in left, right and bilateral SN/VTA, respectively.

**S.6** **Comparison of [^18^F]fallypride BP_ND_ % change values with the literature**

Bearing in mind the differences in stimulant drugs and mode of drug administration (MPH vs. amphetamine (AMPH); oral vs. i.v.), also our regional [^18^F]fallypride BP_ND_ % change values were compared with the ones reported in the aforementioned studies (Figure 5A). It is noteworthy that between-subject variability in our data was systematically smaller compared to the one observed in Slifstein et al 2010 and Cropley et al 2008. The impact of this difference in variance is reflected in the effect sizes illustrated in Figure 5B. The rank order of regional displacement was comparable to Cropley et al 2008 but dissimilar from Slifstein et al 2010, mainly due to differences in BP_ND_ % change in the ventral striatum. One likely explanation for this difference is the mode of drug administration (oral in this paper and in Cropley et al 2008 vs. i.v. in Slifstein et al 2010). The profile of drug concentration over time in brain tissue differs between these two methods, which is likely to have an impact on the time profile of the drug actions across brain regions (reflected by apparent changes in regional [^18^F]fallypride BP_ND_). Thus, it can be concluded that whereas oral and i.v. stimulant doses are similar with regard to their effectiveness in displacing [^18^F]fallypride, the displacement profile across regions associated with each of the administration modes are different.

Figure 5A: Comparison of mean and standard deviation of [^18^F]fallypride BP_ND_ % change following oral MPH (del Campo et al 2012), i.v. AMPH (Slifstein et al 2010) and oral AMPH (Cropley et al 2008). B: Comparison of effects sizes of stimulant drugs on [^18^F]fallypride BP_ND_.


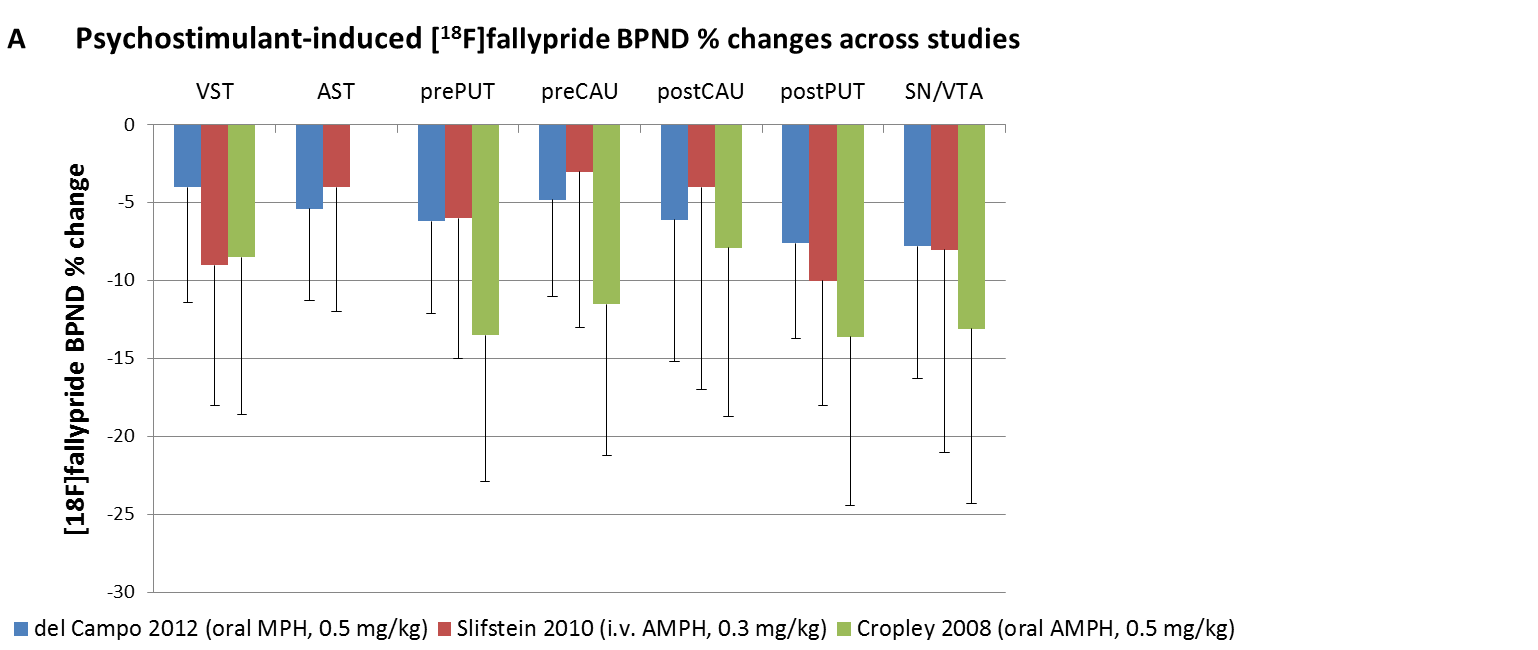


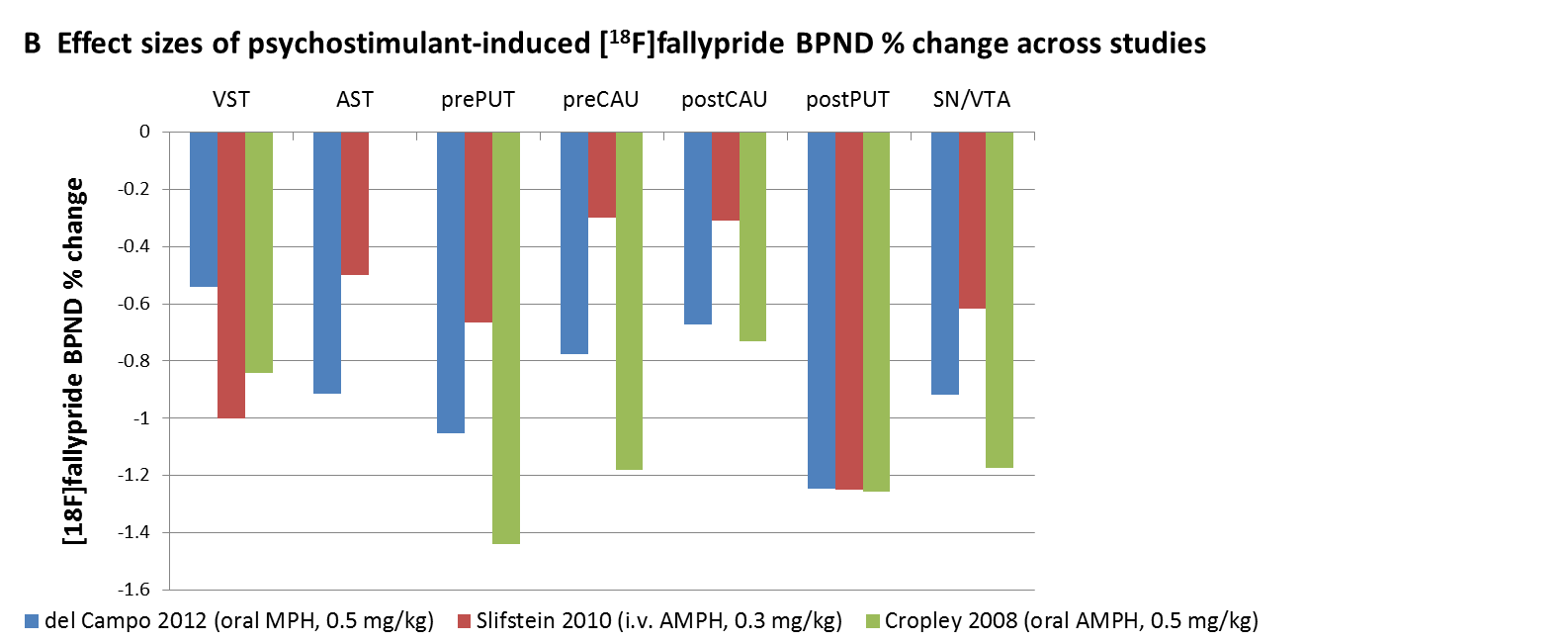


**S.7: Partial volume correction**

Introduction

Partial volume effect (PVES) results from the limited spatial resolution of PET, and results in signal distortions that depend on the tracer distribution, both in the target region and in adjacent tissues . There are two reciprocal effects: Spill-out of activity, i.e. activity originating in an ROI that is recorded outside the ROI, resulting in an underestimation of the signal, and spill-in of signal, i.e. activity originating outside of an ROI that is recorded inside the ROI, leading to an overestimation of the signal. The finding that ADHD patients had reduced grey matter volume in specific brain regions, including the striatum, opened the question as to whether changes in grey matter volume were associated with changes in PVES [^18^F] fallypride BP_ND_ estimation. Thus, PVES of [^18^F] fallypride BP_ND_ were examined across regions, groups and treatment.

It was hypothesised that removing the systematic bias due to PVES would result in significantly greater BP_ND_ values in all regions. This increase was expected as all target ROIs have large boundaries with regions lacking D_2_/D_3_ receptors and hence total spill-out is greater than spill-in. Small regions were expected to suffer the greatest error from PVES and hence increase most following the application of PVC. PVC was also expected to correct for PVES between adjacent striatal sub-regions with different receptor density . Finally, it was hypothesised that the smaller putamen grey matter volume in ADHD patients would be associated with greater PVES.

Method

Partial volume error was corrected in each resliced PET image with an in-house implementation of the method developed by Rousset et al (1998). This method uses high-resolution volumetric MR images coregistered with the PET volumes, to allow segmentation of the images into anatomical regions assumed to have homogeneous tracer uptake. Through knowledge of the point spread function, the method then calculates the contribution that the signal in each region makes to all other regions in a given set of ROIs. Estimates of true activity are determined though inversion of the matrix equation that relates the modelled ROI contributions to the measured PET data. Evidence from phantom studies has shown that this method is capable of providing accurate corrected regional radioactivity concentrations within small structures, including the human basal ganglia .

For implementation of the algorithm, a 1.5 cm thick ellipsoidal region encompassing striatum, thalamus, globus pallidus and SN/VTA was delineated on horizontal sections (Figure 6).


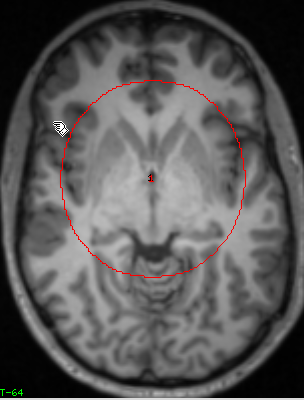


Figure 6: Background region encompassing all ROIs, including striatum and SN/VTA.

This provided a background region of sufficient size relative to the spatial resolution that all spill-out from the structures encompassed by it was measured. Consistency between subjects was achieved by drawing an ellipse 1.5 cm around these structures on the transverse section nearest to the AC-PC line, and copying the ellipse on the 10 slices subsequent to the dorsal and ventral edges of the regions of interest. To avoid errors in PVC propagating from the finite size of the background region, an estimate of the true value in the background region was obtained by drawing two bilateral ellipses in white matter on 10 consecutive slices starting at two slices superior to the ventricles and moving in a dorsal direction. Finally the CSF contained in these background regions was manually delineated on transverse planes using the auto-trace tool in Analyze.

PVC mean ROI values were fed into the simplified reference tissue model to determine regional BP_ND_.

Results

BP_ND_ and BP_ND_ % change values before and after partial volume correction are provided in Table 1.

Table 1: [^18^F]fallypride BP_ND_ before and after partial volume correction. Values are mean (SD).

|  | BP_ND_ (placebo) | | BP_ND_ (MPH) | | BP_ND_ % change | |
| --- | --- | --- | --- | --- | --- | --- |
| ROI | uncorr | PVC | uncorr | PVC | uncorr | PVC |
| VS | 15.1 (1.5) | 26.3 (3.3) | 14.5 (1.6) | 25.5 (4.4) | -4.0 (7.4) | -2.9 (11.7) |
| pre PUT | 18.8 (2.0) | 28.2 (3.4) | 17.6 (1.8) | 26.0 (3.2) | -6.2 (5.9) | -7.5 (8.1) |
| pre CAU | 15.6 (1.8) | 26.3 (3.3) | 14.8 (1.7) | 24.8 (3.4) | -4.8 (6.2) | -5.6 (8.4) |
| post PUT | 17.9 (1.8) | 33.8 (4.1) | 16.5 (1.7) | 30.7 (4.3) | -7.6 (6.1) | -8.9 (8.5) |
| post CAU | 9.9 (1.5) | 29.9 (5.2) | 9.2 (1.5) | 27.3 (5.3) | -6.1 (9.1) | -7.7 (13.5) |
| SN/VTA | 1.4 (0.2) | 4.2 (0.7) | 1.2 (0.2) | 3.8 (0.6) | -7.8 (8.5) | -7.9 (11) |

PVC significantly increased BP_ND_ estimates in all regions (all p < 0.05) (Figure 7). The impact of PVC was greatest in post-commissural caudate and SN/VTA, where PVC almost tripled BP_ND_; these are small regions with a high contrast in signal relative to most/all of the regions that abut them. The striatal sub-region least affected by PVES was the pre-commissural putamen, as expected due to its larger volume. Between-subject variation in BP_ND_ increased when PVC was applied, consistent with the noise-amplifying properties of the Rousset algorithm.

Figure 7: Comparison of regional BP_ND_ values before and after PVC. PVC significantly increased BP_ND_ estimates in all regions (all paired-samples t-tests p<0.05).

Importantly, PVC increased BP_ND_ similarly across groups (Figure 8A) and experimental conditions (Figure 8B) (all p>0.1).

Figure 8: Magnitude of change (in %) between uncorrected and partial volume corrected [^18^F]fallypride BP_ND_ values across ROIs, stratified by group (A) and by treatment (B)


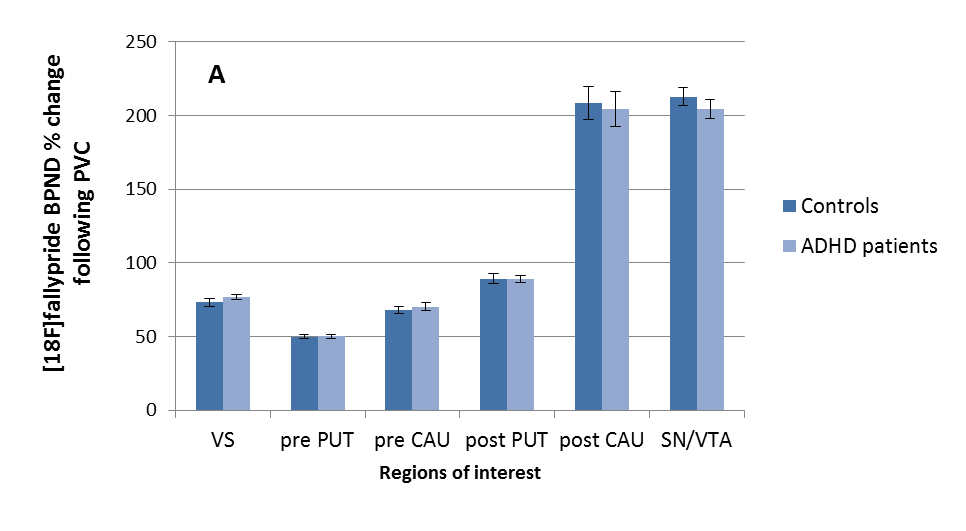


**Figure DF7:** Increases in regional [^18^F]fallypride BP_ND_ estimates were similar A) in ADHD patients and controls and B) across experimental conditions (placebo and methylphenidate).


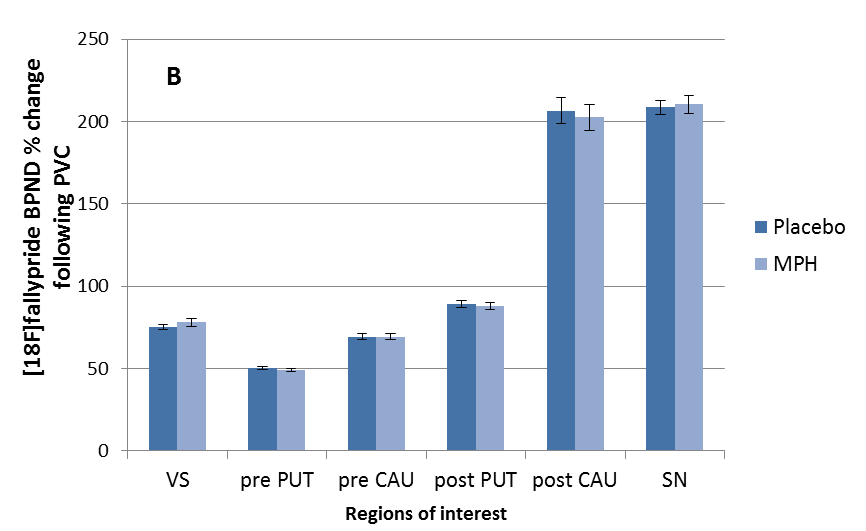


PVC correction resulted in subtle increases of BP_ND_ % change in regions with high BP_ND_ % change (post-commissural putamen and caudate, and pre-commissural putamen), and decreases in regions with low BP_ND_ % change (Figure 9).

Figure 9: MPH-induced [^18^F]fallypride BP_ND_ values before and after PVC. The impact of PVC was only significant in the VS (p=0.033, paired-samples t-test)


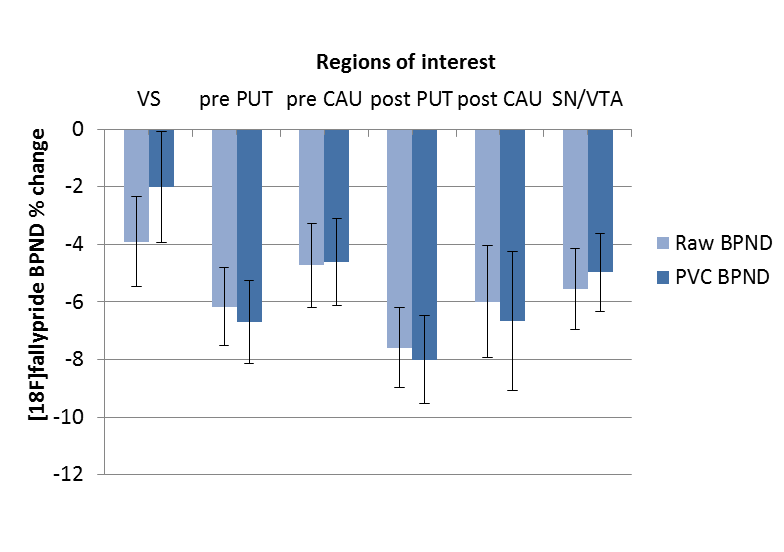


The impact of PVC was only significant in VST (paired samples t-test t(31) = 2.232, p = 0.033), possibly owing to the removal of contamination from the adjacent high-displacement regions (pre-commissural caudate and pre-commissural putamen). As a result, the between-region differences in BP_ND_ % change were further pronounced following PVC and thus, it can be concluded that for adjacent striatal sub-regions which are differentially affected by stimulants, PVC can enhance detection of between-region differences in [^18^F]fallypride BP_ND_ % change. However, note that the rank order of the displacement values across regions remained unchanged.

To conclude, we have shown that the conclusions drawn regarding the DA system in ADHD and the effects of oral MPH were not confounded by PVES. PVC did not have an impact on the main conclusions drawn on the uncorrected BP values.

**References**

Doerge DR, Fogle CM, Paule MG, McCullagh M, Bajic S. Analysis of methylphenidate and its metabolite ritalinic acid in monkey plasma by liquid chromatography/electrospray ionization mass spectrometry. Rapid Commun Mass Spectrom. 2000;14(8):619-23.

Lammertsma AA, Hume SP. Simplified reference tissue model for PET receptor studies. Neuroimage. 1996 Dec;4(3 Pt 1):153-8.

Gunn RN, Lammertsma AA, Hume SP, Cunningham VJ. Parametric imaging of ligand-receptor binding in PET using a simplified reference region model. Neuroimage. 1997 Nov;6(4):279-87.

Cropley VL, Innis RB, Nathan PJ, Brown AK, Sangare JL, Lerner A, et al. Small effect of dopamine release and no effect of dopamine depletion on [18F]fallypride binding in healthy humans. Synapse. 2008 Jun;62(6):399-408.

Slifstein M, Kegeles LS, Xu X, Thompson JL, Urban N, Castrillon J, et al. Striatal and extrastriatal dopamine release measured with PET and [(18)F] fallypride. Synapse. 2010 May;64(5):350-62.

Rousset O, Ma Y, Kamber M, Evans AC. 3D simulations of radiotracer uptake in deep nuclei of human brain. Comput Med Imaging Graph. 1993 Jul-Oct;17(4-5):373-9.

Martinez D, Slifstein M, Broft A, Mawlawi O, Hwang DR, Huang Y, et al. Imaging human mesolimbic dopamine transmission with positron emission tomography. Part II: amphetamine-induced dopamine release in the functional subdivisions of the striatum. J Cereb Blood Flow Metab. 2003 Mar;23(3):285-300.

Rousset OG, Ma Y, Evans AC. Correction for partial volume effects in PET: principle and validation. J Nucl Med. 1998 May;39(5):904-11.
